# Inulin-type fructans and 2’fucosyllactose alter both microbial composition and appear to alleviate stress-induced mood state in a working population compared to placebo (maltodextrin): the EFFICAD Trial, a randomized, controlled trial

**DOI:** 10.1016/j.ajcnut.2023.08.016

**Published:** 2023-08-30

**Authors:** Peter PJ. Jackson, Anisha Wijeyesekera, Claire M. Williams, Stephan Theis, Jessica van Harsselaar, Robert A. Rastall

**Affiliations:** 1 Department of Food and Nutritional Sciences, University of Reading, Reading, United Kingdom; 2 University of Reading, School of Psychology and Clinical Language Science, Reading, United Kingdom; 3 BENEO-Institute, BENEO GmbH, Obrigheim, Germany

**Keywords:** oligofructose, 2’fucosyllactose, gut-brain axis, *Bifidobacterium*, mood state, prebiotics

## Abstract

**Background:**

There is increasing interest in the bidirectional relationship existing between the gut and brain and the effects of both oligofructose and 2’fucosyllactose to alter microbial composition and mood state. Yet, much remains unknown about the ability of oligofructose and 2’fucosyllactose to improve mood state via targeted manipulation of the gut microbiota.

**Objectives:**

We aimed to compare the effects of oligofructose and 2’fucosyllactose alone and in combination against maltodextrin (comparator) on microbial composition and mood state in a working population.

**Methods:**

We conducted a 5-wk, 4-arm, parallel, double-blind, randomized, placebo-controlled trial in 92 healthy adults with mild-to-moderate levels of anxiety and depression. Subjects were randomized to oligofructose 8 g/d (plus 2 g/d maltodextrin); maltodextrin 10 g/d; oligofructose 8 g/d plus 2’fucosyllactose (2 g/d) or 2’fucosyllactose 2 g/d (plus 8 g/d maltodextrin). Changes in microbial load (fluorescence in situ hybridization-flow cytometry) and composition (16S ribosomal RNA sequencing) were the primary outcomes. Secondary outcomes included gastrointestinal sensations, bowel habits, and mood state parameters.

**Results:**

There were significant increases in several bacterial taxa including *Bifidobacterium*, *Bacteroides*, *Roseburia*, and *Faecalibacterium prausnitzii* in both the oligofructose and oligofructose/2’fucosyllactose interventions (all *P* ≤ 0.05). Changes in bacterial taxa were highly heterogenous upon 2’fuscoyllactose supplementation. Significant improvements in Beck Depression Inventory, State Trait Anxiety Inventory Y1 and Y2, and Positive and Negative Affect Schedule scores and cortisol awakening response were detected across oligofructose, 2’fucosyllactose, and oligofructose/2’fucosyllactose combination interventions (all *P* ≤ 0.05). Both sole oligofructose and oligofructose/2’fuscosyllactose combination interventions outperformed both sole 2’fucosyllactose and maltodextrin in improvements in several mood state parameters (all *P* ≤ 0.05).

**Conclusion:**

The results of this study indicate that oligofructose and combination of oligofructose/2’fucosyllactose can beneficially alter microbial composition along with improving mood state parameters. Future work is needed to understand key microbial differences separating individual responses to 2’fucosyllactose supplementation.

This trial was registered at clinicaltrials.gov as NCT05212545.

## Introduction

Modulation of the gut microbiota is a promising way of potentially improving health outcomes. However, cause-and-effect relationships between modulation of gut microbiota and specific health outcomes still remain unclear [1]. Diet is the major driver of gut microbiota composition, and changes can be achieved using functional foods such as prebiotics. Prebiotics, “a substrate that is selectively utilized by host microorganisms conferring a health benefit” [2], include oligofructose (OF) and inulin, which are linear nondigestible carbohydrates inulin-type fructans (ITF) [3]. ITF are the most well substantiated of all prebiotics, with their ability to manipulate microbial composition being demonstrated across a wide array of dosages [[4], [5], [6], [7]].

Additionally, the International Scientific Association for Probiotics and Prebiotics classes several compounds as prebiotic candidates including polyphenols, xylo-oligosaccharides, and human milk oligosaccharides (HMOs), among others [2]. Of all these candidates, HMOs have received rapidly increasing interest and are a class of unconjugated glycans present in breastmilk [8]. Currently, several HMOs are produced commercially including 3’sialyllactose, 6’sialyllactose, lacto-N-tetraose, 3’fucosyllactose, 2’fucosyllactose (2’FL), and lacto-N-neo-tetraose, the most common of these being 2’FL. The ability of the adult microbiota to utilize HMOs remains largely unknown due to the limited number of clinical studies undertaken to date [[9], [10], [11], [12]].

Anxiety and depression are the 2 biggest mental health disorders recorded worldwide, costing health services in excess of 1 trillion USD per year [13,14]. Therefore, there is a demand to find novel approaches to not only treat the burden of disease but also to reduce the burden on the health system [15]. Although the mechanisms by which anxiety and depression are regulated are not well understood [16], there is increasing interest in the bidirectional relationship between the gut and the brain. This gut-brain axis [17] is involved in neuronal development, brain function, and cognitive performance via regulation of neurological, immunological, or endocrine pathways [18].

Within the gut, several genera and species of bacteria can produce several metabolites associated with cognitive state including several neurotransmitters such as γ-aminobutyric acid (GABA), serotonin, and dopamine, as well as short-chain fatty acids (SCFAs) such as acetate, propionate, and butyrate [17,19]. SCFAs produced via saccharolytic fermentation play a role in neurotransmitter production, act as endocrine signaling molecules [19], reduce neuroinflammation via modulation of proinflammatory cytokines [20], and regulate the expression of GABA receptors, enterochromaffin cells, and brain-derived neurotrophic factor and glial-derived neurotrophic factor [21,22].

To date, the ability of prebiotics to improve mood state is still unclear, with studies producing mixed results [[23], [24], [25]]. One area frequently overlooked in such studies is microbial composition. The objective of this double-blind, randomized, placebo-controlled trial, was to investigate the effects of OF and 2’FL, alone and in combination, on microbial load and composition as a primary outcome. As secondary outcomes, we investigated whether OF and 2’FL could improve scores of the Beck Depression Inventory (BDI), State Trait Anxiety Inventory Y1 and Y2 (STAI Y1 and Y2), Positive and Negative Affect Schedule – Short Form (PANAS-SF), and Pittsburgh Sleep Quality Index (PSQI). Saliva and urine samples were collected to assess changes in cortisol awakening response (CAR) and urinary metabolites in adults with mild-to-moderate levels of anxiety and depression.

## Materials and Methods

### Volunteers and recruitment

Healthy adults, both males and females, were recruited from the Reading, Berkshire area of the United Kingdom via previous e-mail lists and by posting on social media. Inclusion criteria were volunteers aged 18 to 50 with BMI ≥ 18.5 and ≤ 30 kg/m^2^, no evidence of gastrointestinal disease, and mild/moderately elevated levels of stress and anxiety as measured via Patient Health Questionnaire-9 (PHQ-9) [26] and Generalized Anxiety Disorder Assessment-7 (GAD-7) [27] (PHQ-9 range: 7–15 and GAD-7 range: 8–16). They were free of food allergies and had a stool frequency of at least 3 bowel movements per week. Exclusion criteria were extreme diets (i.e., ketogenic, vegetarian, vegan, intermittent fasting), antibiotic treatment in the 4 mo preceding the study, anemia, chronic or acute diseases, i.e., (pre)-diabetic. Potential volunteers were also excluded if they had been previously or currently diagnosed with neurological or psychiatric disorders or if they undergone surgical resection of any part of the bowel, were current smokers, and/or had a history of alcohol or drug misuse or if they were pregnant or lactating. Use of laxatives was also not permitted 4 wk prior to beginning of the intervention. Use of antidepressant medication including selective serotonin receptor inhibitors or amitriptyline was not allowed 3 mo prior to commencing the trial.

### Study design and interventions

This was a 4-arm parallel, double-blind, randomized, placebo-controlled trial lasting 5 wk, segregated into a 1-wk run-in and 4-wk intervention phase. The study length (5 wk) was chosen to capture initial day-to-day fluctuations in gastrointestinal habits prior to commencement of intervention, combined with methods previously documented in several mood state and/or gut microbiota supplementation studies [23,24,28,29]. Eligible volunteers were provided with verbal and written study information and gave written informed consent prior to study entry. During the 1-wk run-in period, volunteers were asked to complete a daily bowel habit and gastrointestinal sensation diary to establish baseline values. Subsequently, they were randomly allocated into 1 of 4 intervention groups (*n* = 24) broadly matched for PHQ-9 and GAD-7 scores and sex resulting in an allocation ratio of approximately 3:1 (female:male). Ninety-six adults were divided into Group 1A (19:5), Group 1B (17:7), Group 2A (17:7), and Group 2B (18: 6).

The ITF used was OF (Orafti P95, DP 3-9, average DP 4; BENEO-Orafti). The other test product was 2’FL. 2’FL is an HMO produced commercially using metabolically engineered organisms. 2’FL (96%–98% pure) is a fucosylated HMO composed of L-fucose, D-galactose, and D-glucose and was supplied by BENEO-Orafti. The comparator (placebo) product was maltodextrin, a readily digestible carbohydrate, which consists of varying chains of D-glucose primarily linked by α-(1,4)-linkages of various chain length. To maintain blinding, interventions were packaged in equal weight sachets (sachets A/B = either 8 g OF or maltodextrin and sachets 1/2 = either 2 g maltodextrin or 2’FL) with unique randomization codes by a research assistant not otherwise involved in the study. A random sequence was created utilizing the software RandList Version 1.5. Two grams of 2’FL was the selected dosage based on results of our previous in vitro batch culture fermentations [30], combined with European Food Standards Agency documentation 258/97 stating that 3 g/d of 2’FL is the maximum intended daily intake from food supplements at which no risk of adverse events should occur [31].

Stool, urine, and saliva samples were collected from volunteers as the first urine, stool, and saliva samples after waking at the start (Day 0 [D0]) and the end (Day 28 [D28]) of the intervention phase. Volunteers were also asked to complete self-reported mood state and sleep questionnaires at the start and again at the end of the 4-wk intervention phase. No intervention was given until baseline samples and self-reported questionnaires had been completed. Volunteers were instructed to consume both of their assigned sachets once per day for 4 wk in the morning in water just after or with breakfast resulting in a total daily intervention intake of 10 g. Compliance with consumption of the interventions was assessed by completion of a daily online check-in diary. Participants were considered compliant if they consumed >95% of the supplied supplements. Volunteers were told to not alter their diet or fluid intake during the trial and were asked to record their dietary intake for 3 consecutive days at the start (D0, D1, D2) and end (D26, D27, D28) of the intervention phase into specified diary pages via supplied links. Nutrient intakes (total kcals, protein, fat, saturated fat, total carbohydrates, total sugar, and fiber) were calculated using Nutritics v5.83 [32].

Data were collected and managed using Research Electronic Data Capture (REDCap) electronic data capture tools hosted at the University of Reading [33]. REDCap is a secure, web-based application designed to support data capture for research studies, providing: *1*) an intuitive interface for validated data entry; *2*) audit trails for tracking data manipulation and export procedures; *3*) automated export procedures for seamless data downloads to common statistical packages; and *4*) procedures for importing data from external sources. Blinding was maintained throughout the course of the study, and codes were broken only after completion of data analysis and interpretation.

### Outcome measures

#### Primary outcomes

The primary outcome was differences in *Bifidobacterium* counts as measured by fluorescence in situ hybridization-flow cytometry.

#### Secondary outcomes

##### Bacteriology

Differences in microbial populations between interventions at completion (D28) and change over time from baseline within intervention (D0 compared with D28) as measured by fluorescence in situ hybridization (FISH) flow cytometry (FLOW) and 16S ribosomal RNA (rRNA) sequencing.

##### BDI

The BDI is a 21-question self-reported rating inventory in a multiple-choice format [34]. Within each inventory, volunteers are asked to choose from 1 of 4 statements that best describes their situation in the past 2 wk. Each inventory is scored 0, 1, 2, or 3. 0 represents the normal or least depressive statement and 3 the most depressive statement. An overall score is calculated via summing individual scores for each inventory. Scores range from 0 to 63, lower scores being associated with lower levels of depression.

##### STAI

The STAI is a self-reported questionnaire used for assessing levels of anxiety [35]. The STAI consists of 2 parts, Y1 (State = now/in the moment) and Y2 (Trait = in general). Each form consists of 20 questions, each question being scored from 1 to 4, and each form having a range of 20 to 80. In total, the STAI Y1 and Y2 have 40 questions, with higher scores indicating higher levels of anxiety.

##### PANAS-SF

Current mood (i.e., transient affect) was assessed using the PANAS-SF at D0 and D28 [36]. The PANAS possesses 20 self-reported measures of positive affect (PA; 10 items) and negative affect (NA; 10 items) that can be used on multiple occasions. Each volunteer rated the degree to which they were currently experiencing each item on a 5-point Likert scale. Ratings of positive and negative items were summed to give an overall PA and NA score. Scores range from 10 to 50—higher scores indicate higher levels of PA and NA.

##### PSQI

The PSQI is a self-rating questionnaire consisting of 19 questions plus 5 questions related to either bed partner or roommate. The PSQI assesses sleep quality across 7 different components, each weighted equally, with a score of 0 to 3. Scores from each component are then summed, yielding a total PSQI. Scores range from 0 to 21, with higher scores being associated with poor sleep quality [37].

##### Gastrointestinal sensations, bowel consistency and frequency

Bowel habit and gastrointestinal sensation diaries were completed daily throughout the course of both the 1-wk run-in phase and 4-wk intervention phase in order to assess changes in flatulence, intestinal bloating, abdominal pressure, abdominal pain, and feeling of fullness (all none, mild, moderate, or severe) [4,7,38], and stool frequency and consistency according to the Bristol Stool Form Scale [39]. Any medication use or adverse events were also recorded.

##### Other secondary outcomes

We also collected urine and saliva samples in order to assess changes in urinary metabolites and CAR. Details of sample collection are discussed below. We also collected 3-d consecutive food diary data at D0, D1, and D2 and D26, D27, and D28 of the intervention phase to assess any changes in nutrient intake (total kcals, protein, total fat, saturated fat, total carbohydrates, total sugar, and dietary fiber).

All measures of mood state, gastrointestinal sensation, and bowel habits (consistency and frequency), CAR, and nutrient data were assessed for both differences between interventions at completion (D28) and for change over time from baseline within intervention (D0 compared with D28).

#### Sample collection

##### Fecal samples

Volunteers were provided with sterile stool sample pots for D0 and D28 collections. Freshly collected fecal samples were kept in a 2.5-L Oxoid AnaeroJar with Oxoid AnaeroGen 2.5-L sachet (O_2_ ≤0.1%; CO_2_ 7%–15%). Fecal samples were collected from the volunteer’s place of residence within 2 h of voiding. Samples (1.5 g) for metabolic profiling were stored at −80 C until the study had been completed. An additional 3 g of the same stool sample was diluted 1:10 (w:w) in anaerobic phosphate-buffered saline (PBS, 0.1  M; pH 7.4) and then homogenized using a stomacher (260 paddle beats/min) for 2 min at room temperature. Fecal slurry (20 mL) was then vortexed with 3 mm diameter glass beads for 30 s before being centrifuged at 1500 × *g* for 3 min at room temperature. Seventy-five microliters were then diluted in 675 μL 0.1 M, pH 7.4 PBS (1:100 dilution), and 750-μL aliquots were then stored at −20°C until cells could be fixed. Aliquots were then centrifuged at 11,337 × *g* for 5 min, and the supernatant was discarded. Pellets were then resuspended in 375 μL of 0.1 M, pH 7.4 PBS and fixed in 4% (w:v) paraformaldehyde (1125 μL) for 4 h at 4°C. Fixed cells were centrifuged at 11,337 × *g* for 5 min at room temperature. Samples were then washed with 1 mL PBS, pellets aspirated, and centrifuged at 11,337 × *g* for 5 min. The washing process was repeated twice more. Samples were resuspended in 150 μL PBS and stored in ethanol (1:1, v:v) at −20 °C until analysis via FISH.

##### Urine samples

D0 and D28 midstream urine samples were collected as the first urine sample after waking in sterilized specimen pots. Urine samples were collected from volunteers at the same time as fecal samples. Urine samples were stored at –80°C until analysis by ^1^H-NMR (nuclear magnetic resonance) could be conducted.

##### Salivary cortisol

Hypothalamic-pituitary-adrenal (HPA) activity was assessed at D0 and D28 of the intervention using salivary CAR. Participants were asked to provide 5 saliva samples in 15-mL Falcon tubes. Samples were collected immediately upon waking and subsequently every 15 min until 1 h post waking (0, 15, 30, 45, and 60 min) in separate Falcon tubes. Saliva samples were delivered at the same time as urine and feces. Saliva samples were allocated in 1.5-mL Eppendorf tubes and stored at –80°C until analysis via commercial ELISA (Biotechne, R&D systems) could be completed.

### Enumeration of fecal microbial populations by FISH-FLOW

FISH-FLOW was carried out as previously described [40]. Probes used in this study are listed in Table 1 [[41], [42], [43], [44]]. Fluorescence measures at 488 nm and 640 nm were performed by a BD Accuri C6 Plus . A threshold of 9000 in the forward scatter area and 3000 in the side scatter area was placed to discard background noise, and a gated area was applied in the main density dot to include 90% of the events. Flow rate was 35 μL/min and limit of collection was set for 100,000 events analyzed with Accuri CFlow Sampler software. Bacterial counts were then calculated through consideration of FLOW reading and PBS dilution. The number of log_10_ cells is presented as per gram of wet fresh feces.

**TABLE 1:** Name, sequence, and target group of oligonucleotide probes used in bacterial enumeration

	Sequence (5’ to 3’)	Target groups	Reference
Non-Eub	ACTCCTACGGGAGGCAGC	Control probe complementary to Eub338	[41]
Eub338I	GCTGCCTCCCGTAGGAGT	Most Bacteria	[42]
Eub338II	GCAGCCACCCGTAGGTGT	*Planctomycetales*	[43]
Eub338III	GCTGCCACCCGTAGGTGT	*Verrucomicrobiales*	[43]
Bif164	CATCCGGCATTACCACCC	*Bifidobacterium* spp.	[44]

### Microbial profiling

#### Bacterial DNA extraction

Fresh stool (1.5 g) for metabolic profiling was stored at -80 °C until the study had been completed. Bacterial DNA was extracted from fecal samples using the QIAamp Fast DNA Stool mini kit (QIAGEN) according to the manufacturer’s instructions. In short, fecal samples were homogenized and aliquoted into 2-mL screwcap tubes containing 0.6 g 0.1 mm glass beads. Bead beating was run on a fastprep24 instrument (MP Biomedicals) for 4 cycles of 45 s at speed 4. Two hundred microliters of raw extract were then used for DNA isolation.

#### DNA isolation, library preparation and 16S rRNA gene sequencing

Extracted bacterial DNA was subjected to polymerase chain reaction (PCR) amplification of the V4 region of the 16S rRNA bacterial gene using 2-stage Nextera PCR libraries using the primer pairs 515F (5′- GTG YCA GCM GCC GCG GTA A -3′) and 806R (5′- GGA CTA CNV GGG TWT CTA AT -3′).

Raw sample extracts were diluted to 2.5 ng/μL using Tris buffer, and 5 μL were used in first step PCR, together with 5x HOT FIREPol MultiPlex Mix (Solis BioDyne) and 4 μM primer mix (forward and reverse) 515F/806R (Microsynth). First step PCR samples were purified with NGS Clean Beads (Labgene). Bead ratio was 1:1:2, and beads were washed with 75% ethanol, airdried, and resuspended in Tris buffer. In second step PCR, each sample was individually barcoded, using Nextera XT Index Kit v2 (Illumina, San Diego, California) and 5x HOT FIREPol MultiPlex Mix (Solis BioDyne). Second step PCR samples were purified with NGS Clean Beads (Labgene). The final second step PCR products were quantified using a Quant-iT PicoGreen dsDNA Assay Kit (Thermo Fisher Scientific). Amplicons were pooled in equimolar amounts prior to sequencing. The final pool was quantified using a Quant-iT PicoGreen dsDNA Assay Kit (Thermo Fisher Scientific) and Fragment analyzer (Agilent).

Subsequent PCR libraries were sequenced on an Illumina MiSeq platform using a v2 500 (2×250 bp read length). Pools were diluted to 9.2 pM and loaded together with 15% PhiX (Illumina, FC-110-3001) to increase the diversity of the run resulting in a raw cluster density of 631 and a cluster passed filter rate of 98%. Produced paired-end reads that passed Illumina’s chastity filter were subjected to demultiplexing and trimming of Illumina adaptor residuals using Illumina’s bcl2fastq software version v2.20.0.422. The quality of the reads was checked with the software FastQC version 0.11.8, and sequencing reads that fell below an average Q-score of 20 or had any uncalled bases (N) were removed from further analysis. The locus-specific V4 primers were trimmed from the sequencing reads with the software cutadapt v3.2. Paired-end reads were discarded if the primer could not be trimmed. Trimmed forward and reverse reads of each paired-end read were merged to reform in silico the sequenced molecule considering a minimum overlap of 15 bases using the software USEARCH version 11.0.667. Merged sequences were again quality filtered, allowing a maximum of one expected erroneous base per merged read. Reads that contain ambiguous bases or were outliers regarding the amplicon size distribution were also discarded. Samples that resulted in less than 5000 merged reads were discarded so as not to distort the statistical analysis. Remaining reads were denoised using the UNOISE algorithm implemented in USEARCH to form amplicon sequencing variants (ASVs), discarding singletons and chimeras in the process. The resulting ASV abundance table was then filtered for possible barcode bleed-in contaminations using the UNCROSS algorithm. ASV sequences were compared to the reference sequences of the RDP 16S database provided by https://www.drive5.com/usearch/manual/sintax_downloads.html, and taxonomies were predicted considering a minimum confidence threshold of 0.5 using the SINTAX algorithm implemented in USEARCH. The resulting library was then corrected by taking into consideration numbers of 16S copies and rarefying to an even sampling intensity to reduce bias in diversity metric calculations and quantified as described by Vandeputte et al. [45].

### Metabolic profiling using ^1^H-NMR spectroscopy

Urine samples were thawed for analysis, and a phosphate buffer (pH 7.4 sodium phosphate with 0.2 M disodium phosphate, 0.04 M monosodium phosphate) in deuterium oxide (99.9%) was prepared, with 1 mM 3-(trimethylsilyl) propionic acid-d_4_ sodium salt and 3 mM sodium azide in the solution. Four hundred microliters of each urine sample was mixed with 200 μL buffer. The supernatant was dispensed into 550-μL aliquots to fill 5-mm NMR tubes.

^1^H-NMR spectroscopy analysis was carried out using a Bruker Avance DRX 500 MHz NMR spectrometer (Bruker Biospin). The spectrometer was operated at 500.13 MHz. Urine water spectra were acquired using a standard 1D pulse sequence [recycle delay (RD)-90^◦^-t1-90^◦^-Tm-90^◦^-acquire free induction decay (FID)] with water suppression applied during RD of 2 s, a mixing time of 100 ms and a 90° pulse set at 7.70 μs. Per spectrum, a total of 128 scans were carried out with a spectral width of 14.0019 ppm. The FIDs were multiplied by an exponential function corresponding to 0.3 Hz line broadening. Acquired spectroscopic data were processed using the TopSpin 3.6.5 software package (Bruker Biospin) and nPYc-Toolbox 1.2.7. Further details on the nPYC-Toolbox can be found in Sands et al. [46].

#### Chemometric analysis

Processed spectroscopic data were imported into the SIMCA 17.0 software package (Umetrics AB) to conduct unsupervised and supervised multivariate statistical analysis. Principal component analysis (PCA) was used initially to evaluate similarities/differences in urinary metabolite composition between groups. Principal components were computed to achieve a model that explained most of the variance in the dataset while also having good predictive ability based on the *R*^*2*^ and *Q*^*2*^ model statistics. *R*^*2*^ provides an indication of goodness of fit, and *Q*^*2*^ provides an indication of goodness of prediction. This strategy ensures optimal explanation of variance in the dataset without overfitting the model. PCA was followed by supervised modeling using orthogonal projections to latent structure discriminant analysis (orthogonal partial least square discriminant analysis; OPLS-DA) where the NMR spectroscopic data were the X variables, and the intervention was the Y variable. This analysis maximizes separation between groups in order to focus on the metabolites contributing to this difference. The OPLS-DA models were calculated using 1 predictive component and 2 orthogonal components in order to generate a model with optimal *R*^2^Y (variance explained) and *Q*^2^Y (predictive ability).

### Ethics

The study was given favorable ethical consent by the University of Reading Research Ethics Committee (21/43) and was conducted in accordance with the Declaration of Helsinki. The study was registered at clinicaltrials.gov (NCT05212545). All participants gave written consent prior to study entry.

### Sample size and statistical analysis

The primary outcome measure was bifidobacterial population as log_10_ cells/g wet fecal sample as measured by FISH. It was calculated that to detect a difference in *Bifidobacterium* counts between the 4 interventions, a total of 92 volunteers (*n* = 23 per group) was required. This is based on an 80% probability that the study could detect a 0.5 log_10_ cells/g wet fecal sample difference in colonic bifidobacterial population at a 2-sided 0.05 significance level based on the assumption of a 0.7 log_10_ cells/g wet fecal sample bifidobacteria between subject standard deviation.

Statistical Package for Social Science version 27 (SPSS Inc.) was used for all statistical analyses. Assumptions of normality were assessed using Kolmogorov-Smirnov test, Shapiro-Wilk test, and Q-Q plots. To assess differences between interventions at D28 in bacteriology (FLOW-FISH and quantitative microbiome profiling [QMP]), mood state (BDI, STAI, and PANAS-SF), PSQI, CAR, bowel habits, gastrointestinal sensation, and nutrient data, a general linear analysis of covariance (ANCOVA) was performed using intervention as a fixed factor, D28 values as dependent variables, and D0 values, sex, and PHQ-9 and GAD-7 scores as covariates. General linear ANCOVA with intervention as a fixed factor was also applied for bowel habits, gastrointestinal sensation, and nutrient data whereby either D26 to D28 (for nutrient data) or final week scores (for bowel habit and gastrointestinal sensation) were the dependent variables, with D0 to D2 (for nutrient data) run-in week (for bowel habit and gastrointestinal sensation), sex, and PHQ-9 and GAD-7 scores as covariates. Marginal general linear models (MGLMs) were used to assess changes over time (D0 compared with D28) for mood state, PSQI, CAR, and nutrient data as previously described [[47], [48], [49]]; repeated measures general linear models were applied for changes over time in bacteriology. All pairwise comparisons within each ANCOVA, MGLM, and repeated measures general linear analysis of variance were corrected for type 1 errors using Bonferroni adjustment.

Correlations between bacterial taxa and mood state were assessed employing fold change ((post-pre)/pre) using nonparametric Spearman’s rank correlation corrected for using false discovery rate (FDR). All tests were 2-tailed and *P* values ≤ 0.05 were considered statistically significant. Graphs were generated in GraphPad Prism version 10.0.0 for Windows (GraphPad Software).

## Results

### Participant data

**FIGURE 1:**
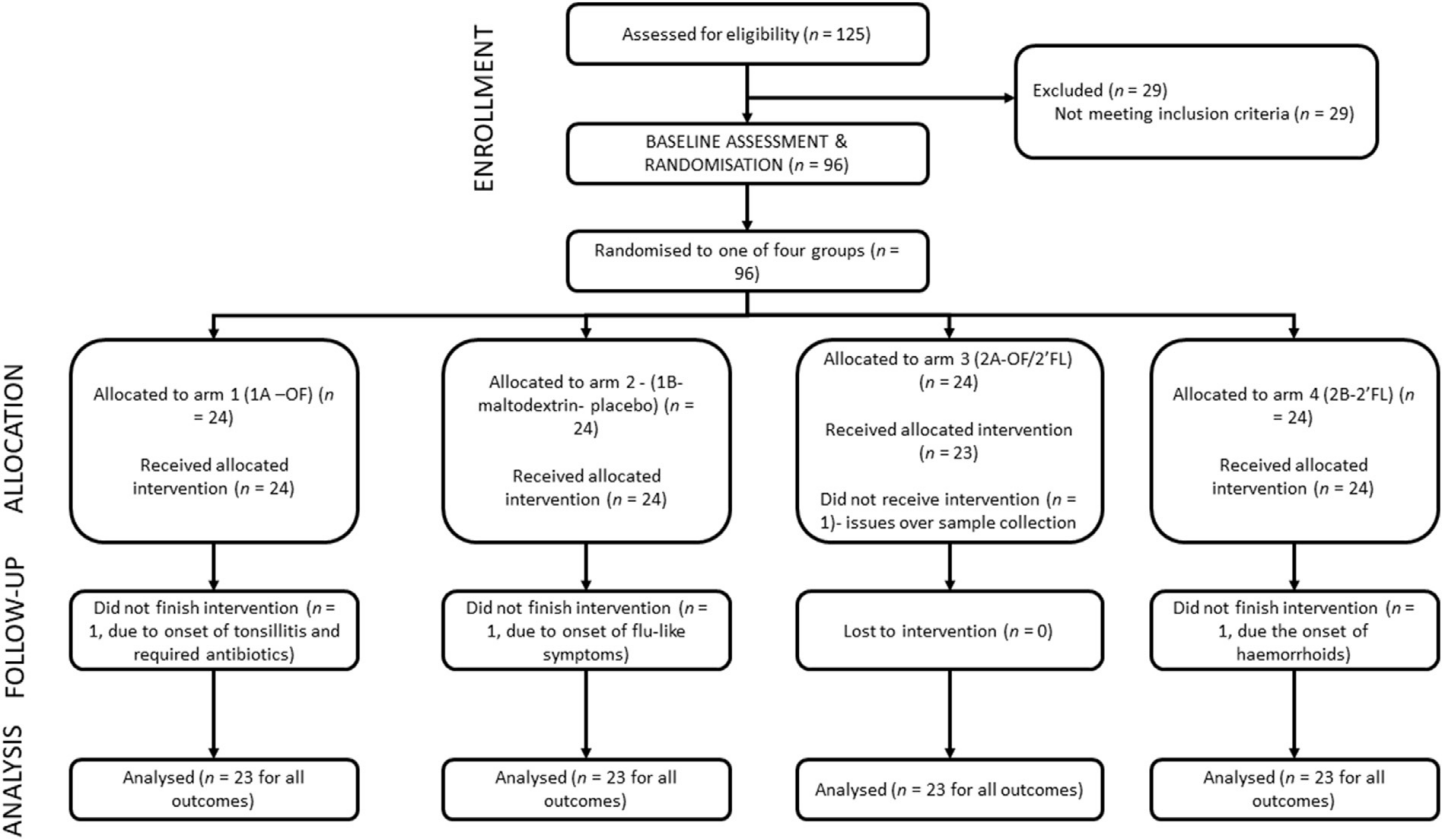
CONSORT diagram of participant flow through the intervention. Abbreviations: 2’FL, 2’fucosyllactose; OF, oligofructose; OF/2’FL, oligofructose/2’fucosyllactose.

In total, 125 volunteers expressed interest and were screened for eligibility, of whom 96 were randomized (*n* = 24 in each group). Of these, 4 volunteers withdrew from the trial; 92 volunteers completed the trial (62 females and 30 males) (*n* = 23 in each group) and were included in analysis for all primary and secondary outcomes (Figure 1).

Table 2 reports baseline subject characteristics (*n* = 23 per group) (age, height, weight, and BMI), mean and range segregated by intervention. Mean subject age was 28.13 y, weight 66.92 kg, height 168.08 cm, and BMI 23.53 kg/m^2^.

**TABLE 2:** Baseline participant characteristics

Intervention	OF (*n* = 23)	Maltodextrin (*n* = 23)	OF/2’FL (*n* = 23)	2’FL (*n* = 23)	Cohort (overall) (*n* = 92)
Age (y)	29.17 (19–50)	29.04 (20–47)	25.39 (19–47)	28.91 (19–46)	28.13 (19–50)
Weight (kg)	65.98 (53.34–105.00)	69.23 (50.00–94.00)	67.95 (48.20–100.00)	64.53 (46.00–93.50)	66.92 (46.00–105.00)
Height (cm)	169.80 (157.00–196.00)	167.70 (145.00–193.00)	168.50 (154.00–187.00)	166.30 (155.00–181.00)	168.08 (145.00–196.00)
BMI (kg/m^2^)	22.79 (18.72–29.07)	24.41 (19.02–28.73)	23.74 (19.84–29.96)	23.17 (18.59–29.73)	23.53 (18.59–29.96)

Mean and range segregated by intervention (*n* = 23 per group) and total cohort (*n* = 92). Abbreviations: 2’FL, 2’fucosyllactose; OF, oligofructose; OF/2’FL, oligofructose/2’fucosyllactose.

### Dietary intake

Nutrient data at baseline in the first week (D0, D1, and D2) and final week (D26, D27, and D28) of the intervention are presented in Table 3. No significant differences were detected in either total energy, protein, carbohydrates, total sugar, fat, or saturated fat either within or between interventions (all *P* ≥ 0.05). Analysis of 3-d food diaries at baseline revealed dietary fiber intakes were estimated at 19.66 g/d (Table 3).

**TABLE 3:**
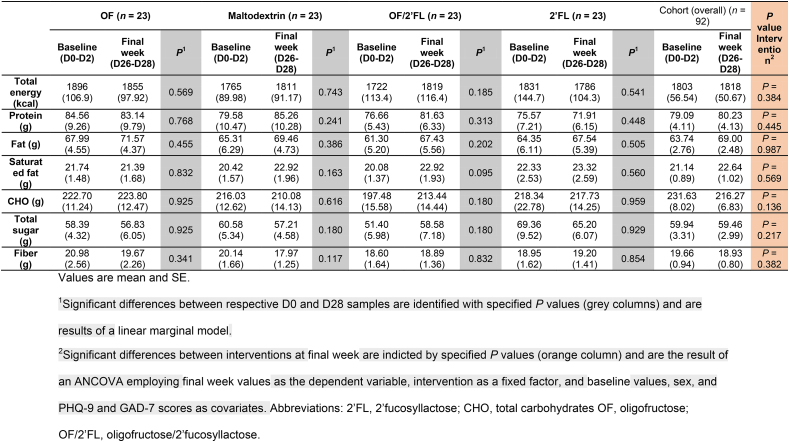
Energy and nutrient intake at baseline (D0–D2) and at completion (D26–D28) of intervention phase in 92 volunteers (*n* = 23 per group)

### Bacterial enumeration by FISH-FLOW

**FIGURE 2:**
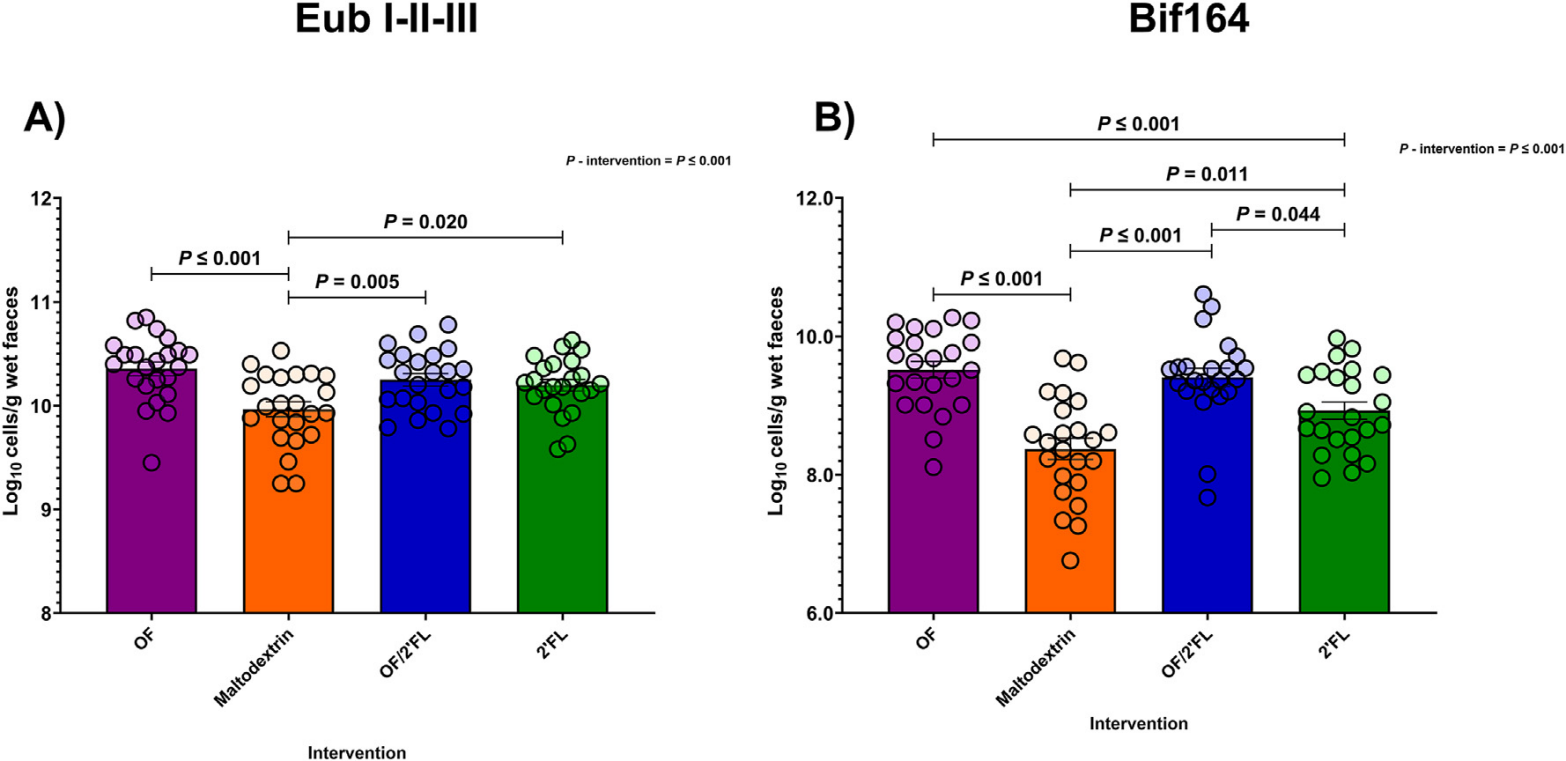
Group differences in bacterial groups measured by FISH-FLOW (log_10_ cells/g wet feces) at D28 of the intervention using probes: Total bacteria (Eub I-II-III) (A) and *Bifidobacterium* spp. (Bif164) (B) in 92 adults (*n* = 23 per group). Values are mean and (SE) (all points) expressed as log_10_ cells/g wet feces. ANCOVA was used to calculate intervention effect employing intervention as a fixed factor, D28 values as the dependent variable, and D0 values, sex, and PHQ-9 and GAD-7 scores as covariates. Results that are statistically significant between interventions are displayed by specified *P* values. Abbreviations: 2’FL, 2’fucosyllactose; OF, oligofructose; OF/2’FL, oligofructose/2’fucosyllactose.

Ninety-two volunteers provided stool samples at baseline and the end of the intervention. Figure 2 and Supplemental Table 1 report changes in total (Eub I,II,III) and (Bif164) *Bifidobacterium* counts observed across the 4 intervention groups between D0 and D28 of the intervention using FISH-FLOW.

As shown in Figure 2A**,** ANCOVA analysis revealed significant differences between intervention at D28 in Eub I,II,III counts with highest counts documented in OF, OF/2’FL, and 2’FL interventions [F (3,84) = 9.89, *P* ≤ 0.001; η^2^: 0.261] (Figure 2A). Specifically, pairwise comparisons revealed Eub I,II,III counts were significantly higher in OF, OF/2’FL, and 2’FL interventions compared with maltodextrin—OF (*P* ≤ 0.001; mean difference: 0.36; 95% confidence interval [CI]: 0.18, 0.54), OF/2’FL (*P* = 0.005; mean difference: 0.23; 95% CI: 0.05, 0.41), and 2’FL (*P* = 0.020; mean difference: 0.20; 95% CI: 0.02, 0.38) (Figure 2A). Repeated measures analysis revealed significant increases from baseline in Eub I,II,III counts were detected in OF (*P* ≤ 0.001: 0.35 mean difference), OF/2’FL (*P* ≤ 0.001; 0.22 mean difference) and 2’FL (*P* ≤ 0.001; 0.19 mean difference) interventions, but not maltodextrin (*P* = 0.972) (Supplemental Table 1).

Similarly, regarding Bif164 (*Bifidobacterium* spp.), ANCOVA revealed that significant differences between interventions at D28 in Bif164 counts and were highest at completion in OF, OF/2’FL, and 2’FL interventions [F (3,84) = 20.52, *P* ≤ 0.001; η^2^: 0.423]. Post hoc pairwise comparisons revealed Bif164 counts were significantly higher in the OF (*P* ≤ 0.001; mean difference: 1.13; 95% CI: 0.71, 1.56), OF/2’FL (*P* ≤ 0.001; mean difference: 0.94; 95% CI: 0.52, 1.36), and 2’FL (*P* = 0.011; mean difference: 0.51; 95% CI: 0.82, 0.93) interventions compared with maltodextrin (Figure 2B). In addition, Bif164 counts were also significantly higher in both OF (*P* ≤ 0.001; mean difference: 0.62; 95% CI: 0.20, 1.05) and OF/2’FL (*P* = 0.044; mean difference: 0.43; 95% CI: 0.01, 0.86) interventions compared with only 2’FL. Finally, repeated measures analysis revealed significant increases from baseline in Bif164 counts in OF (*P* ≤ 0.001; 0.95 mean difference), OF/2’FL (*P* ≤ 0.001; 0.72 mean difference), and 2’FL (*P* = 0.016; 0.30 mean difference) interventions, but not maltodextrin (*P* = 0.146; −0.18 mean difference) (Supplemental Table 1).

### Microbiota profiling analysis—QMP

**FIGURE 3:**
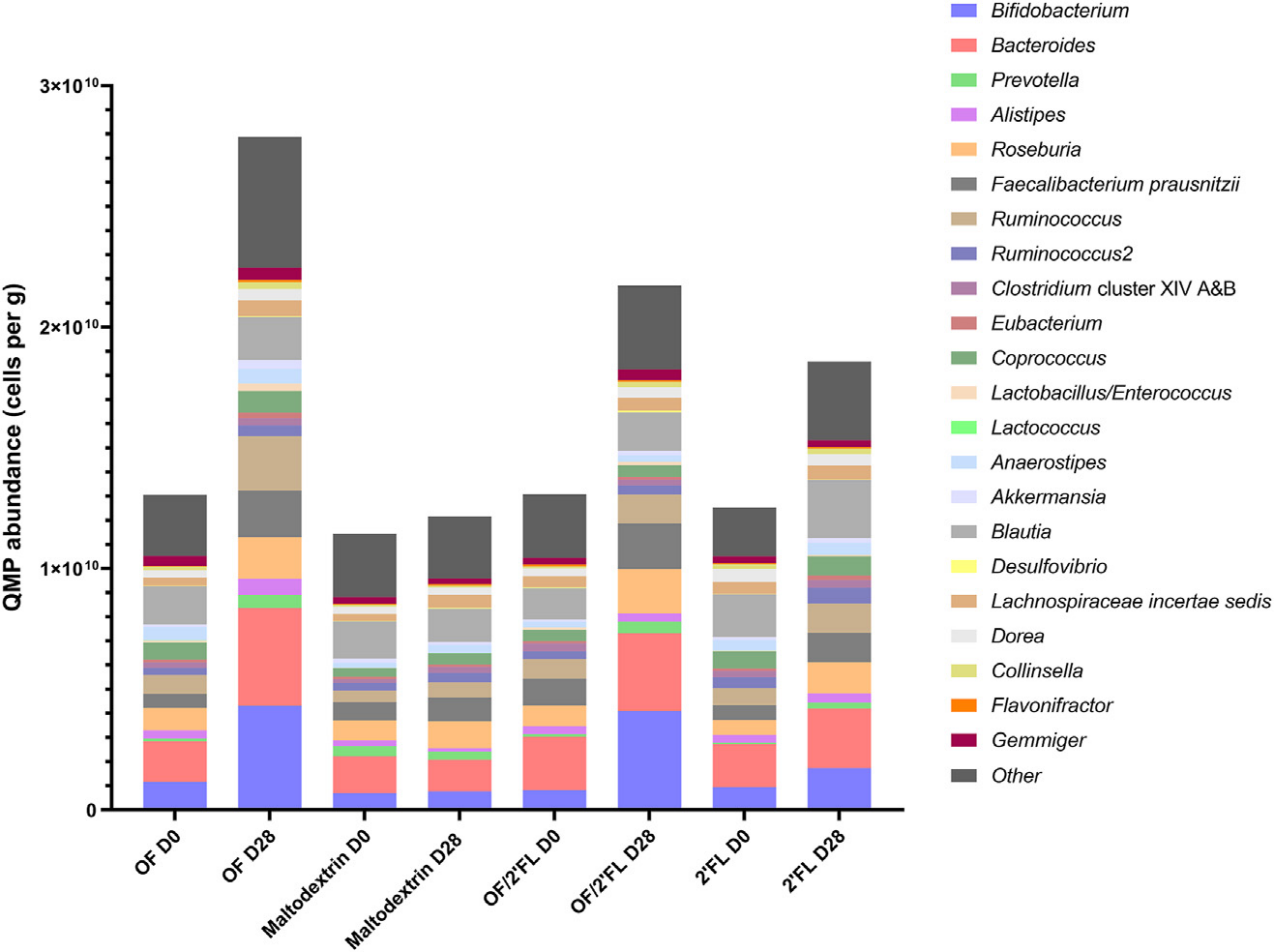
Quantitative microbiome profiling (QMP) data of overall 16S rRNA sequencing data recorded across all 4 interventions at D0 and D28. Numbers are expressed as cells/g feces. Abbreviations: 2’FL, 2’fucosyllactose; OF, oligofructose; OF/2’FL, oligofructose/2’fucosyllactose.

**FIGURE 4:**
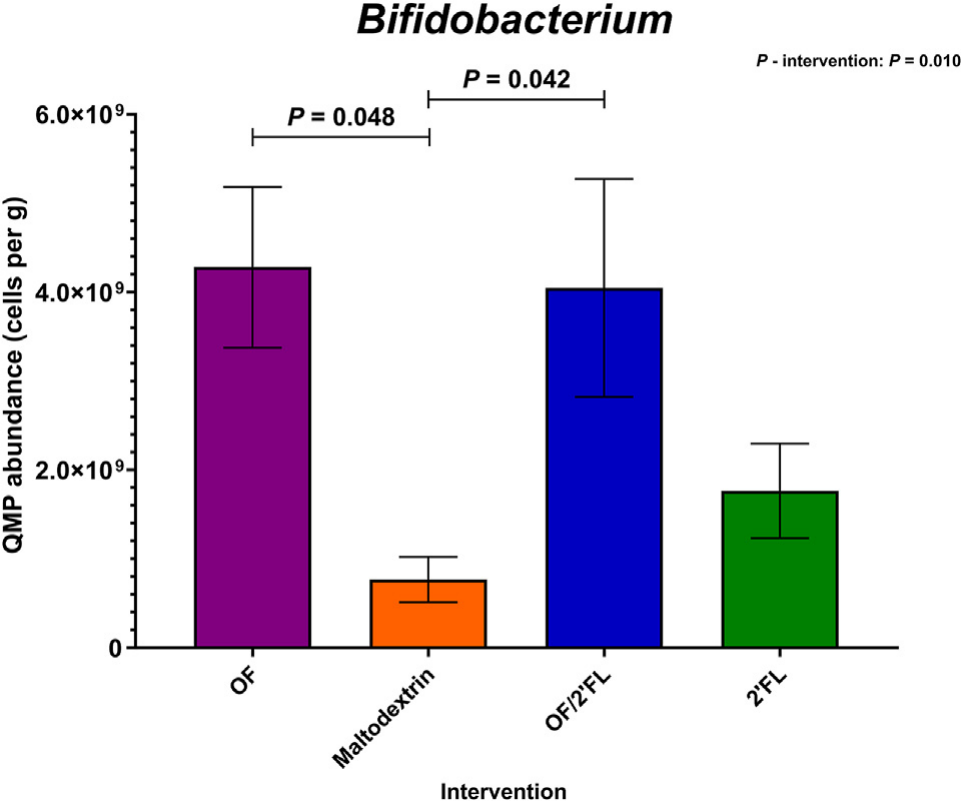
Quantitative microbiome profiling data of group differences in *Bifidobacterium* 16S rRNA sequencing data observed at completion (D28) of the intervention phase in 92 adults (*n* = 23 per group). Mean and SE expressed as numbers of cells/g feces. ANCOVA was used to calculate intervention effect employing intervention as a fixed factor, D28 values as the dependent variable, and D0 values, sex, and PHQ-9 and GAD-7 scores as covariates. Results that are statistically significant between interventions are displayed by specified *P* values. Abbreviations: 2’FL, 2’fucosyllactose; OF, oligofructose; OF/2’FL, oligofructose/2’fucosyllactose.

**FIGURE 5:**
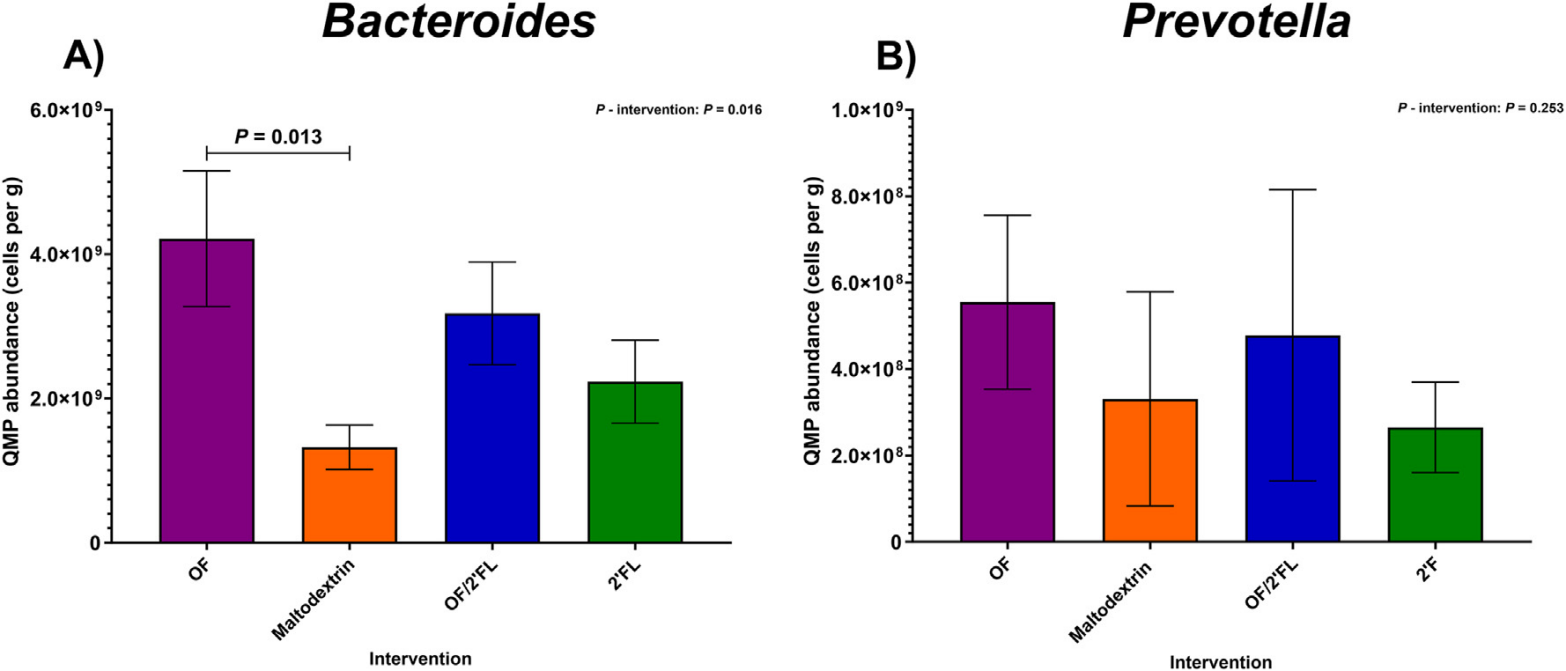
Quantitative microbiome profiling data of group differences in *Bacteroides* (A) and *Prevotella* (B) 16S rRNA sequencing data observed at completion (D28) of the intervention phase in 92 adults (*n* = 23 per group). Mean and SE expressed as numbers of cells/g feces. ANCOVA was used to calculate intervention effect employing intervention as a fixed factor, D28 values as the dependent variable, and D0 values, sex, and PHQ-9 and GAD-7 scores as covariates. Results that are statistically significant between interventions are displayed by specified *P* values. Abbreviations: 2’FL, 2’fucosyllactose; OF, oligofructose; OF/2’FL, oligofructose/2’fucosyllactose;

**FIGURE 6:**
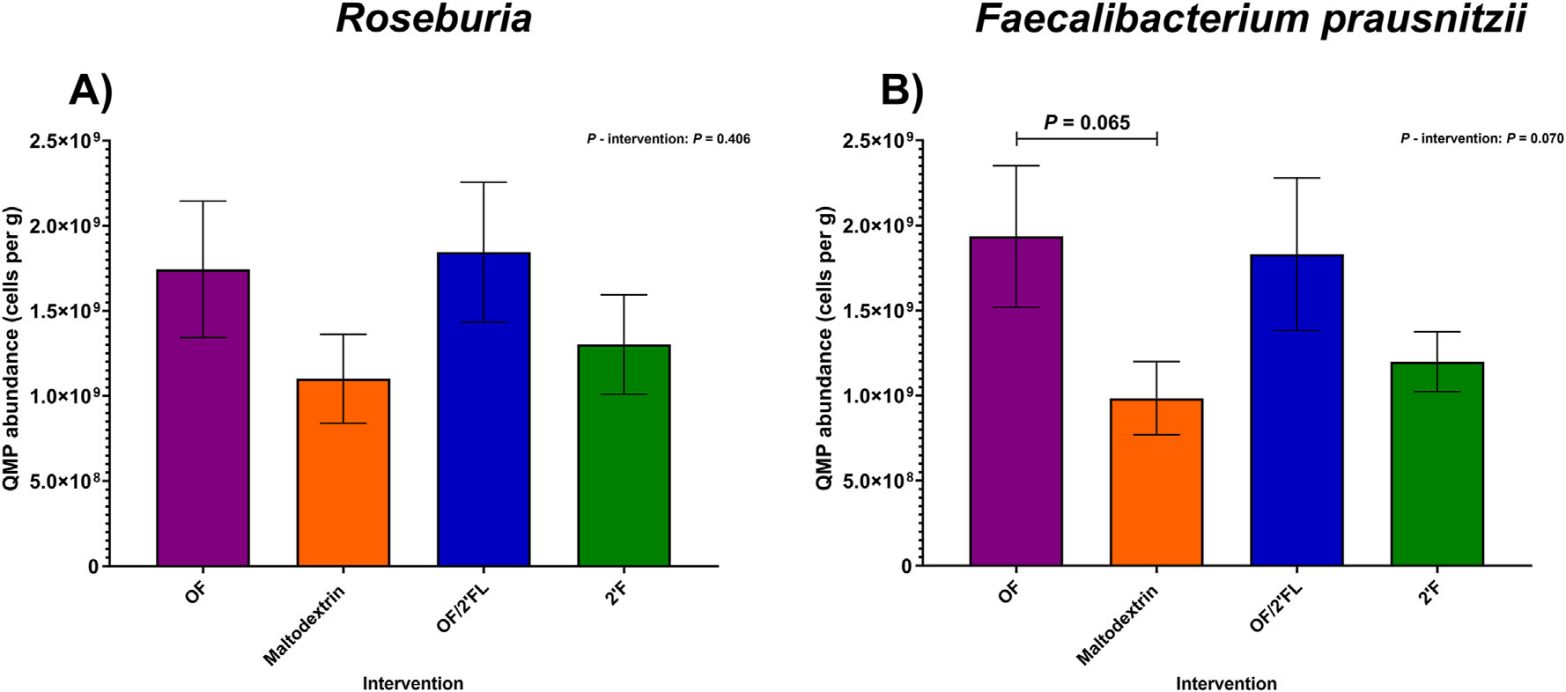
Quantitative microbiome profiling data of group differences in *Roseburia* (A) and *Faecalibacterium prausnitzii* (B) sequencing data observed at completion (D28) of the intervention phase in 92 adults (*n* = 23 per group). Mean and SE expressed as numbers of cells/g feces. ANCOVA was used to calculate intervention effect employing intervention as a fixed factor, D28 values as the dependent variable, and D0 values, sex, and PHQ-9 and GAD-7 scores as covariates. Results that are statistically significant between interventions are displayed by specified *P* values. Abbreviations: 2’FL, 2’fucosyllactose; OF, oligofructose; OF/2’FL, oligofructose/2’fucosyllactose.

Figure 3 reports the overall QMP microbial abundance data. Figures 4, **5**, and **6** report the most significant changes documented from the QMP data across the 4 interventions at completion.

At the phylum level, there were several differences detected on completion of the intervention. The magnitude of change varied substantially between interventions. Regarding *Actinomycetota* (*Actinobacteria*), ANCOVA revealed numbers to be highest in both OF and OF/2’FL interventions at D28 [F (3,84) = 4.63, *P* = 0.005; η^2^: 0.142]. Post hoc pairwise comparisons revealed numbers of *Actinomycetota* to be significantly higher in both OF (*P* = 0.024; mean difference: 3.54 × 10^9^; 95% CI: 3.09 × 10^8^, 6.78 × 10^9^) and OF/2’FL (*P* = 0.028; mean difference: 3.43 × 10^9^; 95% CI: 2.42 × 10^8^, 6.62 × 10^9^) compared to maltodextrin. Subsequent repeated measures analysis revealed significant increases from baseline in *Actinomycetota* counts in both OF (*P* ≤ 0.001; 3.44 × 10^9^ mean difference) and OF/2’FL (*P* ≤ 0.001; 3.45 × 10^9^ mean difference) interventions.

Other notable changes at the phylum level occurred in *Bacteroidota* (*Bacteroidetes*), with ANCOVA revealing a significant intervention effect [F (3,84) = 3.98, *P* = 0.012; η^2^: 0.122] with numbers of *Bacteroidota* being highest in the only OF intervention at completion. Post hoc analysis indicated that numbers of *Bacteroidota* (*Bacteroidetes*) in the OF intervention were significantly higher compared with maltodextrin at completion (*P* = 0.007; mean difference: 3.68 × 10^9^; 95% CI: 7.09 × 10^8^, 6.67 × 10^9^). Repeated measures analysis revealed that numbers of *Bacteroidota* (*Bacteroidetes*) only significantly increased from baseline in the OF intervention, increasing by an average of 3.32 × 10^9^ cells/g wet feces (*P* ≤ 0.001).

Similarly, regarding *Bacillota* (*Firmicutes*), ANCOVA reported a small intervention effect [F (3,84) = 3.43, *P* = 0.021; η^2^: 0.109] with numbers again being highest in the only OF intervention at completion. This was confirmed with post hoc comparisons revealing numbers of *Bacillota* in the OF intervention being significantly higher compared with maltodextrin only (*P* = 0.013; mean difference: 6.30 × 10^9^; 95% CI: 9.32 × 10^8^, 1.17 × 10^10^). Repeated measures analysis revealed that *Bacillota* counts significantly increased from baseline in OF (*P* ≤ 0.001; 7.64 ×10^9^ mean difference), OF/2’FL (*P* = 0.008; 3.79 × 10^9^ mean difference) and 2’FL (*P* = 0.004; 4.10 × 10^9^ mean difference), but not maltodextrin intervention (*P* = 0.386; 1.20 × 10^9^ mean difference) (Supplemental Table 2).

At the genus level, microbial responses varied significantly among the interventions, with the largest changes documented in *Bifidobacterium*, *Bacteroides*, *Prevotella*, *Roseburia*, and *Faecalibacterium prausnitzii*.

Regarding *Bifidobacterium,* ANCOVA revealed *Bifidobacterium* counts to be highest in both OF and OF/2’FL combination intervention at completion as indicated by a significant intervention effect [F (3,83) = 4.05, *P* = 0.010; η^2^: 0.128]. Post hoc pairwise comparisons revealed *Bifidobacterium* counts in both OF (*P* = 0.048; mean difference: 3.20 × 10^9^; 95% CI: 1.52 × 10^7^, 6.39 × 10^9^) and OF/2’FL (*P* = 0.042; mean difference: 3.23 × 10^9^; 95% CI: 7.57 × 10^7^, 6.38 × 10^9^) interventions were significantly higher compared with maltodextrin. Subsequent repeated measures analysis identified that *Bifidobacterium* counts significantly increased from baseline in both the OF (*P* ≤ 0.001; 3.12 × 10^9^ mean difference) and OF/2’FL (*P* ≤ 0.001; 3.23 × 10^9^ mean difference) interventions, but not 2’FL (*P* = 0.314; 8.23 × 10^8^ mean difference) or maltodextrin (*P* = 0.898; 7.28 × 10^7^ mean difference) (Figure 4 and Supplemental Table 2).

A significant intervention effect was also detected in *Bacteroides* at completion [F (3,83) = 3.64, *P* = 0.016; η^2^: 0.116] with numbers of *Bacteroides* being highest in the OF intervention only at completion. Post hoc pairwise comparisons revealed *Bacteroides* counts to be significantly higher in the OF intervention compared with maltodextrin (*P* = 0.013; mean difference: 2.87 × 10^9^; 95% CI: 4.11 × 10^8^, 5.33 × 10^9^) (Figure 5A). Repeated measures analysis revealed significant increases from baseline in *Bacteroides* counts in the OF intervention only (*P* ≤ 0.001; 2.53 × 10^9^ mean difference) (Supplemental Table 2).

QMP results for *Prevotella* are documented in Figure 5B. No intervention effect was observed [F (3,83) = 1.38, *P* = 0.253; η^2^: 0.048]. Consequently, repeated measures analysis revealed significant increases in the number of *Prevotella* from baseline in both OF (*P* = 0.013; 4.43 × 10^8^ mean difference) and OF/2’FL (*P* = 0.039; 3.66 × 10^8^ mean difference) interventions only (Figure 5B and Supplemental Table 2).

As shown in Figure 6A, no significant intervention effect was observed at completion in *Roseburia* counts [F (3,83) = 0.98, *P* = 0.406; η^2^: 0.034]. Using repeated measures analysis, significant increases from baseline in *Roseburia* counts was documented in OF (*P* = 0.008; 8.03 × 10^8^ cells/g mean difference), OF/2’FL (*P* = 0.001; 9.85 × 10^8^ cells/g mean difference), and 2’FL (*P* = 0.026; 6.77 × 10^8^ cells/g mean difference) interventions, but not maltodextrin (*P* = 0.332; 2.98 × 10^8^ mean difference) (Supplemental Table 2).

Regarding *Faecalibacterium prausnitzii*, a trend toward differences in bacterial counts was observed between interventions at completion [F (3,83) = 2.44, *P* = 0.070; η^2^: 0.081] (Figure 6B). This was confirmed with pairwise comparisons identifying a trend toward significantly higher *Faecalibacterium prausnitzii* count in the OF intervention compared to maltodextrin (*P* = 0.065; mean difference: 1.08 × 10^9^; 95% CI: −3.98 × 10^7^, 2.20 × 10^9^). Finally, repeated measures analysis revealed *Faecalibacterium prausnitzii* counts significantly increased from baseline in the OF (*P* ≤ 0.001; 1.35 × 10^9^ mean difference), OF/2’FL (*P* = 0.015; 7.11 × 10^8^ mean difference), and 2’FL (*P* = 0.047; 5.80 × 10^8^ mean difference) interventions, but not maltodextrin (*P* = 0.898; 2.26 × 10^8^ mean difference) (Supplemental Table 2).

A number of significant within group-increases were also recorded for the OF intervention in bacterial taxa including *Alistipes* (*P* = 0.004), *Ruminococcus* (*P* ≤ 0.001), *Lactobacillus/Enterococcus* (*P* = 0.029), *Eubacterium* (*P* = 0.003), *Akkermansia* (*P* = 0.015), *Desulfovibrio* (*P* = 0.021), *Lachnospiraceae incertae sedis* (*P* = 0.004), *Flavonifractor* (*P* = 0.011), and *Collinsella* (*P* = 0.007). Significant changes in *Collinsella* (*P* = 0.006), *Desulfovibrio* (*P* = 0.017) and *Gemmiger* (*P* = 0.033) were also detected in the OF/2’FL intervention (Supplemental Table 2). An increase in *Ruminococcus2* (*P* = 0.040) and *Eubacterium* (*P* = 0.016) were the only other significant changes detected in the 2’FL intervention. There was also a large increase in *Blautia* seen in the 2’FL intervention; however, due to the high level of heterogeneity seen between individuals, only a trend toward significance was recorded (*P* = 0.063) (Supplemental Table 2).

### Bowel habit and function

**FIGURE 7:**
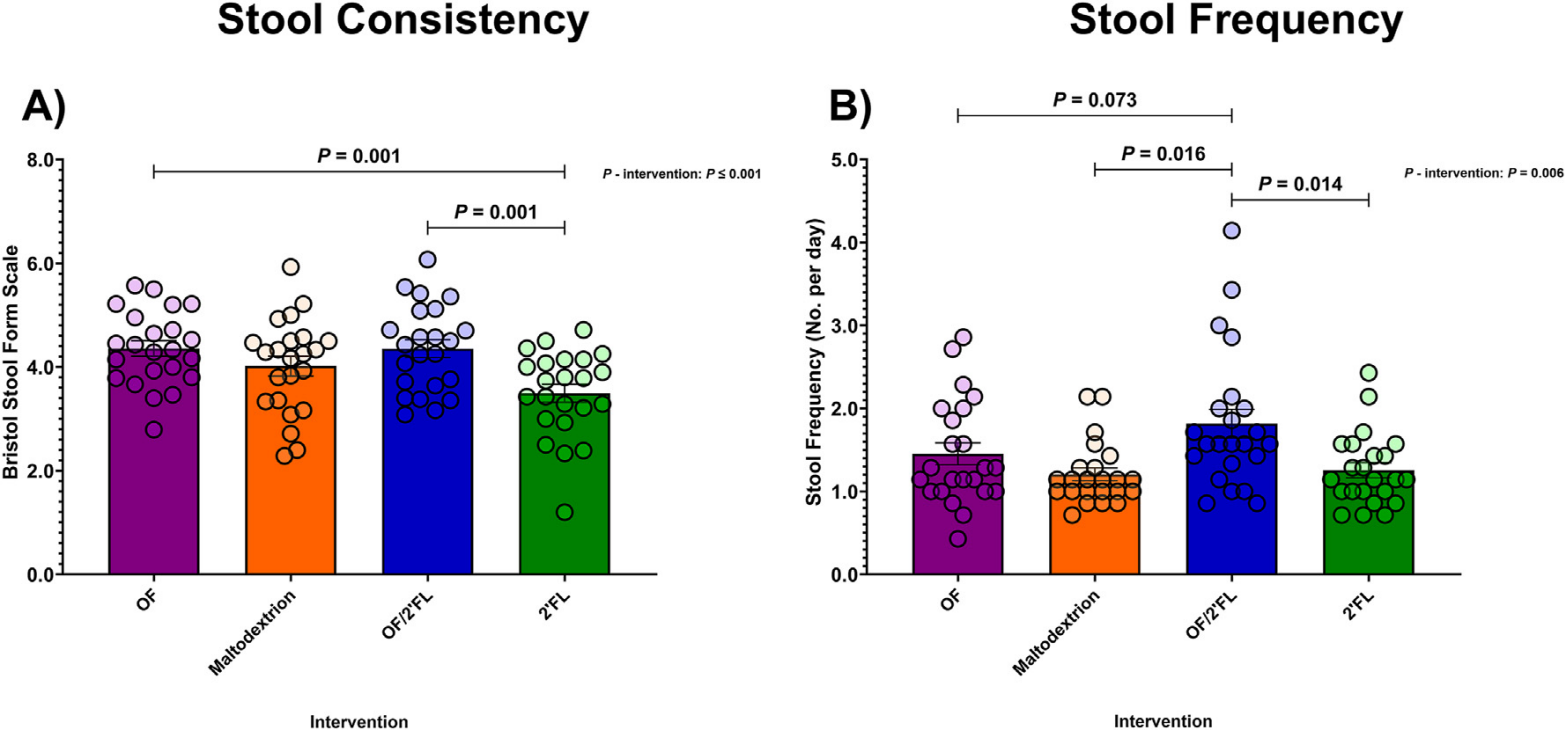
Group differences in gastrointestinal scores for stool consistency as per the Bristol Stool Form Scale (A) and Stool Frequency (B) observed at last week of intervention (D22–D28 average) in 92 volunteers (*n* = 23 per group). Mean and SE (all volunteer points). ANCOVA was used to calculate intervention effect employing intervention as a fixed factor, final week D22–D28 average values as the dependent variable, and run-in week values, sex, and PHQ-9 and GAD-7 scores as covariates. Results that are statistically significant between interventions are displayed by specified *P* values. Abbreviations: 2’FL, 2’fucosyllactose; OF, oligofructose; OF/2’FL, oligofructose/2’fucosyllactose.

Changes in gastrointestinal sensations (flatulence, intestinal bloating, abdominal pressure, abdominal pain, and feeling of fullness), stool consistency, and stool frequency were self-recorded daily throughout both the 1-wk run-in and 28-d intervention period. Scores of 0, 1, 2, and 3 corresponded to none, mild, moderate, and severe [4,7,38]. Data are presented as an average of the last week of the intervention phase (D22–D28). Changes in stool consistency measured as per Bristol Stool Form Scale [39] and stool frequency are reported in Figure 7.

There was no effect of intervention detected in any gastrointestinal sensation (flatulence, intestinal bloating, abdominal pressure, abdominal pain, or feeling of fullness [Supplemental Table 3]). Using repeated measures analysis, small increases in both flatulence (*P* = 0.001; 0.31 mean difference) and intestinal bloating (*P* = 0.007; 0.26 mean difference) scores were detected in the OF group on completion. However, as stated, neither of these increases were significantly higher and not differentiated compared to any of the other interventions (flatulence [F (3,84) = 1.42, *P* = 0.242; η^2^: 0.048] or intestinal bloating [F (3,84) = 2.16, *P* = 0.098; η^2^: 0.072]) and were rated as none to mild at most.

There was a significant difference between interventions for stool consistency (trends toward stool softness) with higher scores documented at D28 for both the OF and OF/2’FL interventions [F (3,84) = 6.60, *P* ≤ 0.001; η^2^: 0.191]. Post hoc pairwise comparisons revealed stool consistency scores to be higher in both the OF (*P* = 0.001; mean difference: 0.79; 95% CI: 0.23, 1.35) and OF/2’FL (*P* = 0.001; 0.80 mean difference; 95% CI: 0.24, 1.38) interventions compared to only 2’FL (Figure 7A). Importantly, MGLM revealed significant increases in stool consistency scores from baseline in both OF (*P* = 0.006; 0.48 mean difference) and OF/2’FL (*P* = 0.007; 0.46 mean difference) interventions (Supplemental Table 3).

Regarding changes in stool frequency, ANCOVA revealed a significant difference between interventions with highest scores documented in the OF/2’FL combination [F (3,84) = 4.42, *P* = 0.006; η^2^: 0.136]. As shown in Figure 7B, increases in stool frequency scores in the OF/2’FL intervention were significantly higher compared to both maltodextrin (*P* = 0.016; mean difference: 0.44; 95% CI: 0.06, 0.83) and 2’FL (*P* = 0.014; 0.44 mean difference; 95% CI: 0.06, 0.83). There was also a trend toward higher stool frequency scores in the OF/2’FL intervention compared with only OF (*P* = 0.073; 0.36 mean difference; 95% CI: −0.02, 0.74) (Figure 7B). Finally, MGLM analysis identified stool frequency scores significantly increased from baseline in the OF/2’FL combination intervention only (*P* ≤ 0.001; 0.37 mean difference) (Supplemental Table 3).

### Mood state

#### BDI

**FIGURE 8:**
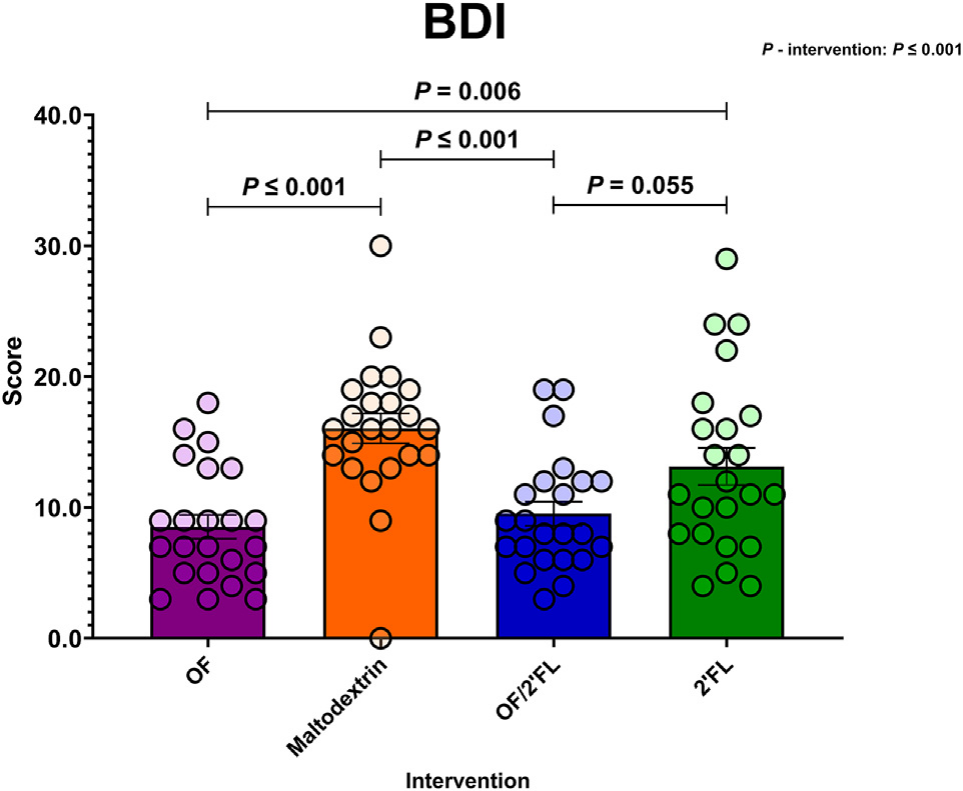
Group differences in Beck Depression Inventory (BDI) scores observed at D28 across the 4 interventions in 92 volunteers (*n* = 23 per group). Mean and SE (all volunteer points). ANCOVA was used to calculate intervention effect employing intervention as a fixed factor, D28 values as the dependent variable, and D0 values, sex, PHQ-9 and GAD-7 scores as covariates. Results that are statistically significant between interventions are displayed by specified *P* values. Abbreviations: 2’FL, 2’fucosyllactose; OF, oligofructose; OF/2’FL, oligofructose/2’fucosyllactose.

Figure 8 displays BDI scores across the 4 interventions at D28. At the end of the study period, ANCOVA revealed a significant difference in BDI scores **between** interventions [F (3,84) = 11.86, *P* ≤ 0.001; η^2^: 0.298]. Pairwise comparisons revealed BDI scores were significantly lower in the OF and OF/2’FL interventions compared with maltodextrin: OF (*P* ≤ 0.001; mean difference: −7.96; 95% CI: −12.03, −3.90); OF/2’FL (*P* ≤ 0.001; mean difference: −6.86; 95% CI: −10.93, −2.80). The OF intervention also showed significantly lower BDI scores at completion compared with 2’FL (*P* = 0.006; mean difference: −5.12; 95% CI: −9.18, −1.05) (Figure 8 and Supplemental Table 4). There was also a trend toward significantly lower BDI scores in the OF/2’FL intervention group compared to 2’FL (*P* = 0.055; −4.02 mean difference; 95% CI: −8.09, 0.06). Importantly, MGLM analysis documented a significant reduction in BDI scores from D0 to D28 in OF (*P* ≤.001; −10.44 mean difference), OF/2’FL (*P* ≤ 0.001; −9.57 mean difference) and 2’FL (*P* ≤ 0.001; −4.70 mean difference), but not maltodextrin (*P* = 0.074; −2.00 mean difference).

#### STAI Y1 and Y2

**FIGURE 9:**
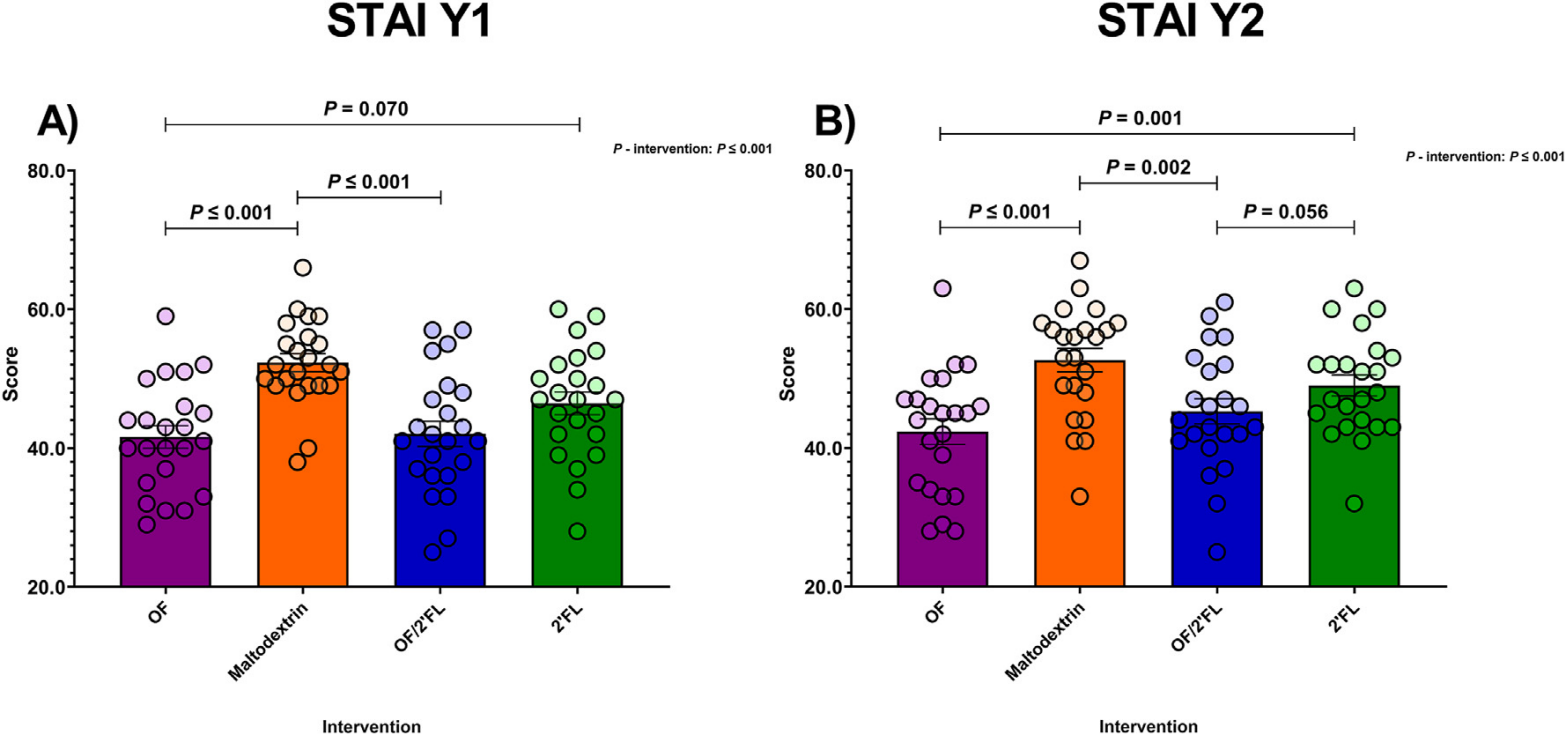
Group differences in State Trait Anxiety Inventory (STAI) Y1 (State) (A) and Y2 (Trait) (B) scores observed at D28 across the 4 interventions in 92 volunteers (*n* = 23 per group). ANCOVA was used to calculate intervention effect employing intervention as a fixed factor, D28 values as the dependent variable, and D0 values, sex, and PHQ-9 and GAD-7 scores as covariates. Results that are statistically significant between interventions are displayed by specified *P* values. Abbreviations: 2’FL, 2’fucosyllactose; OF, oligofructose; OF/2’FL, oligofructose/2’fucosyllactose.

Significant comparisons for STAI Y1 and STAI Y2 are displayed in Figure 9. Regarding STAI Y1 results, ANCOVA revealed a significant effect of intervention [F (3,84) = 9.81, *P* ≤ 0.001; η^2^: 0.259]. As shown in Figure 9A, the lowest STAI Y1 scores were seen in the OF, OF/2’FL, and 2’FL groups (Figure 9A). Post hoc pairwise comparisons revealed that the OF and OF/2’FL interventions recorded significantly lower STAI Y1 scores compared with maltodextrin: OF (*P* ≤ 0.001; mean difference: −11.38; 95% CI: −17.69, −5.07); OF/2’FL (*P* ≤ 0.001; mean difference: −10.06; 95% CI: −16.36, −3.76) at completion. There was also a trend toward lower STAI Y1 scores in the OF intervention compared with 2’FL (*P* = 0.070; mean difference: −6.05; 95% CI: −12.39, 0.29) (Figure 9A). Analyzing the change over the 28 d of the intervention, significant reductions in STAI Y1 scores in the OF (*P* ≤.001; −13.30 mean difference), OF/2’FL (*P* ≤ 0.001; −13.31 mean difference) and 2’FL (*P* = 0.001; −6.48 mean difference) interventions were seen (Supplemental Table 4). No change was apparent in the group receiving maltodextrin (*P* = 0.063; −3.61 mean difference).

Similarly, analysis of STAI Y2 scores revealed a significant difference between interventions [F (3,84) = 10.73, *P* ≤ 0.001; η^2^: 0.277], with pairwise comparisons revealing STAI Y2 scores to be significantly lower in both the OF and OF/2’FL interventions compared to maltodextrin: OF (*P* ≤ 0.001; mean difference: −10.69; 95% CI: −16.44, −4.94); OF/2’FL (*P* = 0.002; mean difference: −8.04; 95% CI: −13.80, −2.29). STAI Y2 scores were also significantly lower in the OF intervention compared with 2’FL (*P* = 0.001; mean difference: −8.40; 95% CI: −14.21, −2.59) at completion (Figure 9B). Furthermore, there was also a trend toward significantly lower STAI Y2 scores in the OF/2’FL intervention group compared with 2’FL (*P* = 0.056; −5.75 mean difference; 95% CI: −11.59, 0.056). Finally, MGLM analysis revealed that STAI Y2 scores were significantly reduced from baseline in OF (*P* ≤ 0.001; −13.35 mean difference), OF/2’FL (*P* ≤ 0.001; −10.74 mean difference) and 2’FL (*P* ≤ 0.001; −4.52 mean difference) interventions, but not maltodextrin (*P* = 0.086; −2.52 mean difference) (Supplemental Table 4).

#### PANAS-SF

**FIGURE 10:**
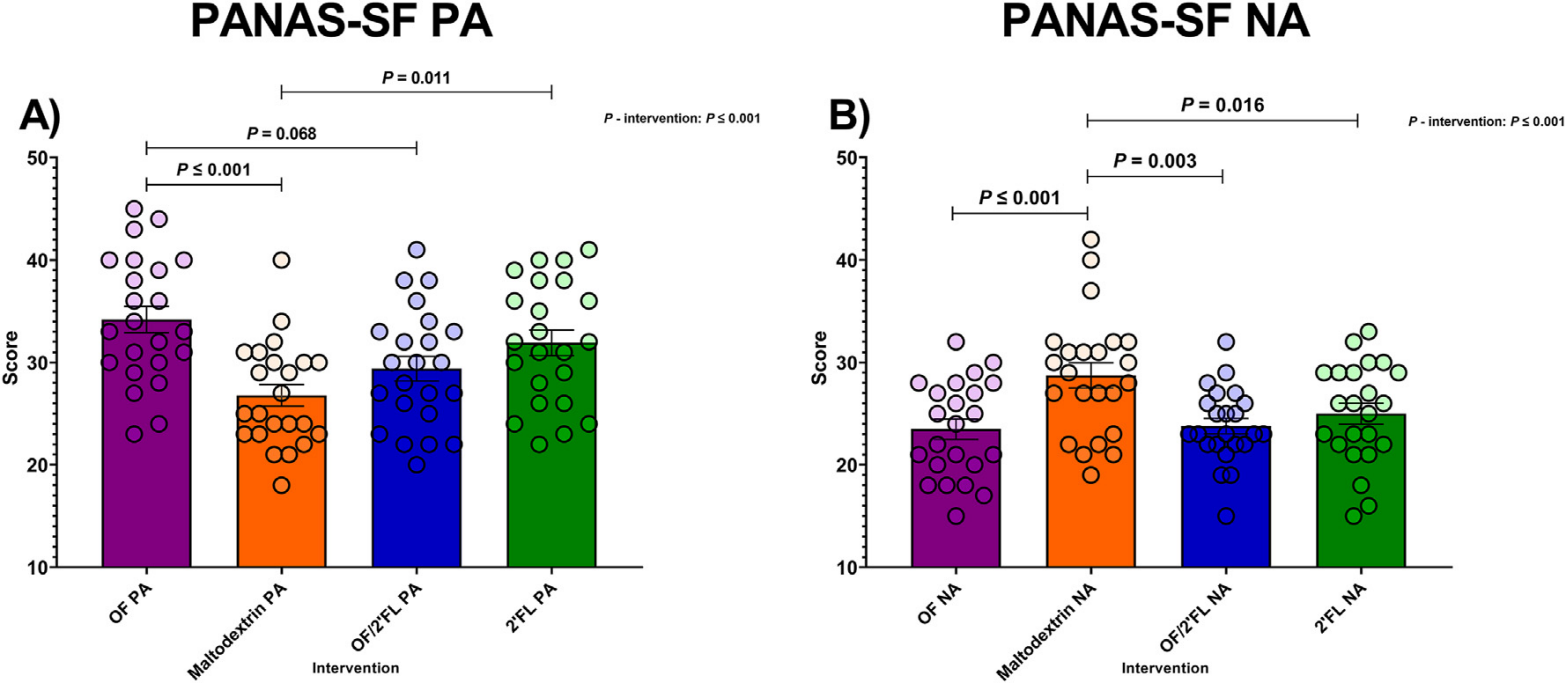
Group differences in Positive affect (PA) (A) and Negative affect (NA) (B) Schedule – Short Form scores observed at D28 across the 4 interventions in 92 volunteers (*n* = 23 per group). Mean and SE (all volunteer points). ANCOVA was used to calculate intervention effect employing intervention as a fixed factor, D28 values as the dependent variable, and D0 values, sex, and PHQ-9 and GAD-7 scores as covariates. Results that are statistically significant between interventions are displayed by specified *P* values. Abbreviations: 2’FL, 2’fucosyllactose; OF, oligofructose; OF/2’FL, oligofructose/2’fucosyllactose.

Significant PA and NA scores are reported in Figure 10. Regarding PA scores, ANCOVA revealed a significant intervention [F (3,84) = 6.87, *P* ≤ 0.001; η^2^: 0.197]. Post hoc analysis documented PA scores to be significantly higher in both sole OF and 2’FL interventions compared with maltodextrin at D28: OF (*P* ≤ 0.001; mean difference: 7.05; 95% CI: 2.56, 11.55); 2’FL (*P* = 0.011; mean difference: 5.33; 95% CI: 0.84, 9.81). Additionally, there was also a trend toward significantly higher PA scores in the OF intervention compared with the OF/2’FL combination (*P* = 0.068; mean difference: 4.32; 95% CI: −0.19, 8.83) at completion (Figure 10A and Supplemental Table 4). MGLM analysis revealed PA scores significantly increased from baseline to D28 in OF (*P* ≤ 0.001; 7.87 mean difference), OF/2’FL (*P* ≤ 0.001; 4.78 mean difference), and 2’FL (*P* ≤ 0.001; 7.43 mean difference) interventions, but not maltodextrin (*P* = 0.176; 1.87 mean difference) (Supplemental Table 4).

In terms of NA scores, ANCOVA revealed significant differences between interventions [F (3,84) = 8.30, *P* ≤ 0.001; η^2^: 0.229]. Subsequent post hoc analysis revealed NA scores at D28 were significantly lower for the OF, OF/2’FL, and 2’FL treatment groups compared with maltodextrin: OF (*P* ≤ 0.001; mean difference: −6.46; 95% CI: −10.16, −2.77), OF/2’FL (*P* = 0.003; mean difference: −4.88; 95% CI: −8.48, −1.28), and 2’FL (*P* = 0.016; mean difference: −4.13; 95% CI: −7.75, −0.52) (Figure 10B). Finally, our MGLM analysis revealed NA scores significantly decreased (reflecting less negative mood) from baseline in the OF (*P* ≤ 0.001; −8.61 mean difference), OF/2’FL (*P* ≤ 0.001; −4.87 mean difference), and 2’FL (*P* ≤ 0.001; −4.96 mean difference) interventions, but not maltodextrin (*P* = 0.878; −1.74 mean difference) (Supplemental Table 4).

#### PSQI

**FIGURE 11:**
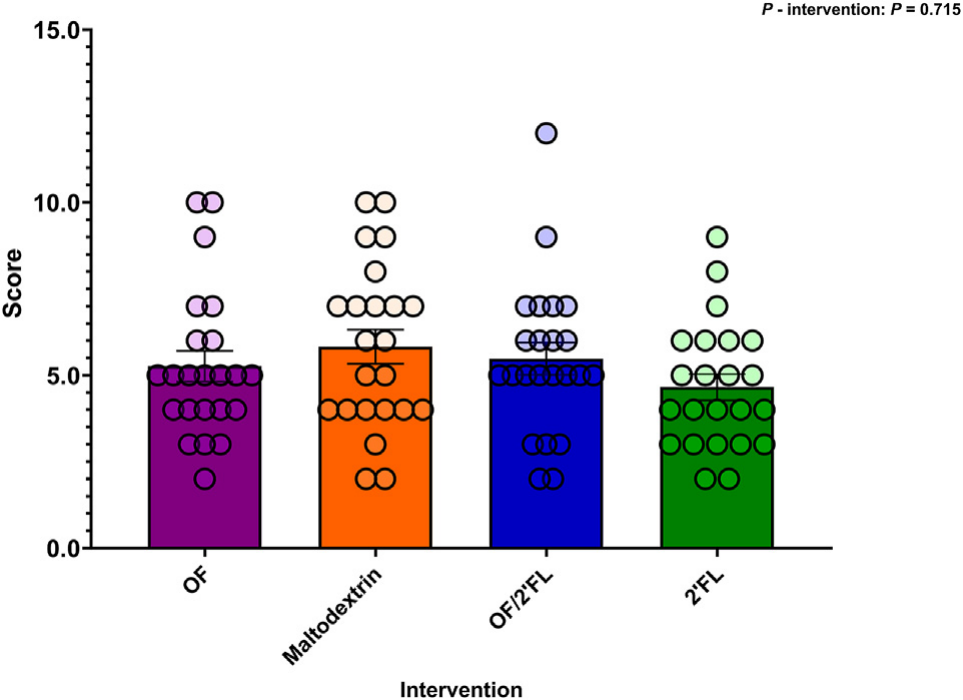
Differences in Pittsburgh Sleep Quality Index (PSQI) scores observed at D28 across the 4 interventions in 92 volunteers (*n* = 23 per group). Mean and SE (all volunteer points). ANCOVA was used to calculate intervention effect employing intervention as a fixed factor, D28 values as the dependent variable, and D0 values, sex, and PHQ-9 and GAD-7 scores as covariates. Results that are statistically significant between interventions are displayed by specified *P* values. Abbreviations: 2’FL, 2’fucosyllactose; OF, oligofructose; OF/2’FL, oligofructose/2’fucosyllactose.

There was no effect of intervention on PSQI scores at completion [F (3,84) = 0.454, *P* = 0.715; η^2^: 0.16] (Figure 11). Subsequently, repeated measures analysis revealed significant reductions in PSQI scores from baseline to D28 (reflecting better sleep) across all 4 interventions: OF (*P* ≤ 0.001; −1.35 mean difference), 2’FL (*P* ≤ 0.001; −1.35 mean difference), maltodextrin (*P* =0.004; −1.08 mean difference), and OF/2’FL (*P* = 0.003; −1.13 mean difference) (Supplemental Table 4).

#### CAR

**FIGURE 12:**
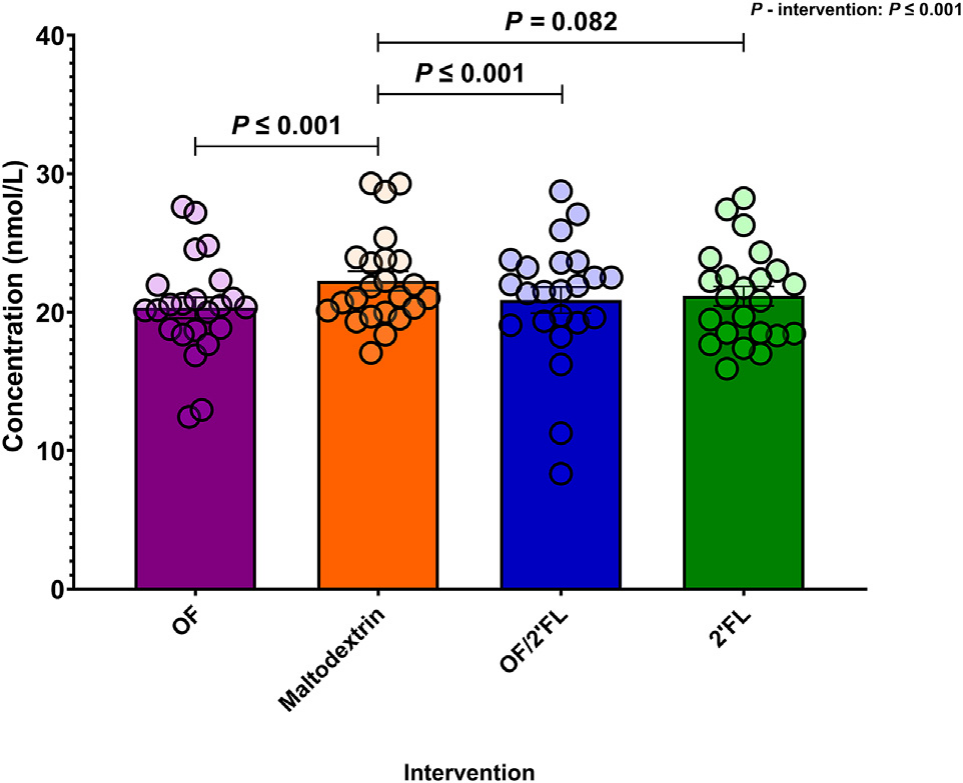
Group differences in total average cortisol awakening response (0, 15, 30, 45, and 60 min) values (ng/mL) observed at D28 across the 4 interventions in 92 volunteers (*n* = 23 per group). Mean and SE (all volunteer points). ANCOVA was used to calculate intervention effect employing intervention as a fixed factor, D28 values as the dependent variable, and D0 values, sex, and PHQ-9 and GAD-7 scores as covariates. Results that are statistically significant between interventions are displayed by specified *P* values. Abbreviations: 2’FL, 2’fucosyllactose; OF, oligofructose; OF/2’FL, oligofructose/2’fucosyllactose.

Figure 12 reports the average total CAR in nmol/L at D28 across the 4 intervention groups. Analysis revealed a significant intervention interaction at completion [F (3,84) = 8.83, *P* ≤ 0.001; η^2^: 0.240] with OF and OF/2’FL interventions documenting lowest CAR values. Post hoc pairwise comparisons indicated that both the OF and OF/2’FL interventions recorded significantly lower CAR values at D28 in comparison with maltodextrin: OF (*P* ≤ 0.001; mean difference: −2.33; 95% CI: −3.73, −0.93) and OF/2’FL (*P* ≤ 0.001; mean difference: −2.27; 95% CI: −3.68, −0.87). There was also a trend toward significantly lower CAR values in the 2’FL intervention, again compared with maltodextrin (*P* = 0.082; mean difference: −1.31; 95% CI: −2.71, 0.95) at D28. Finally, MGLM analysis revealed CAR values significantly decreased from baseline in the OF (*P* ≤ 0.001; −1.99 mean difference), OF/2’FL (*P* ≤ 0.001; −1.93 mean difference), and 2’FL (*P* = 0.008; −0.96 mean difference) interventions, but not maltodextrin (*P* = 0.300; 0.37 mean difference) (Supplemental Table 4).

### Correlation between bacteriology and mood state

**FIGURE 13:**
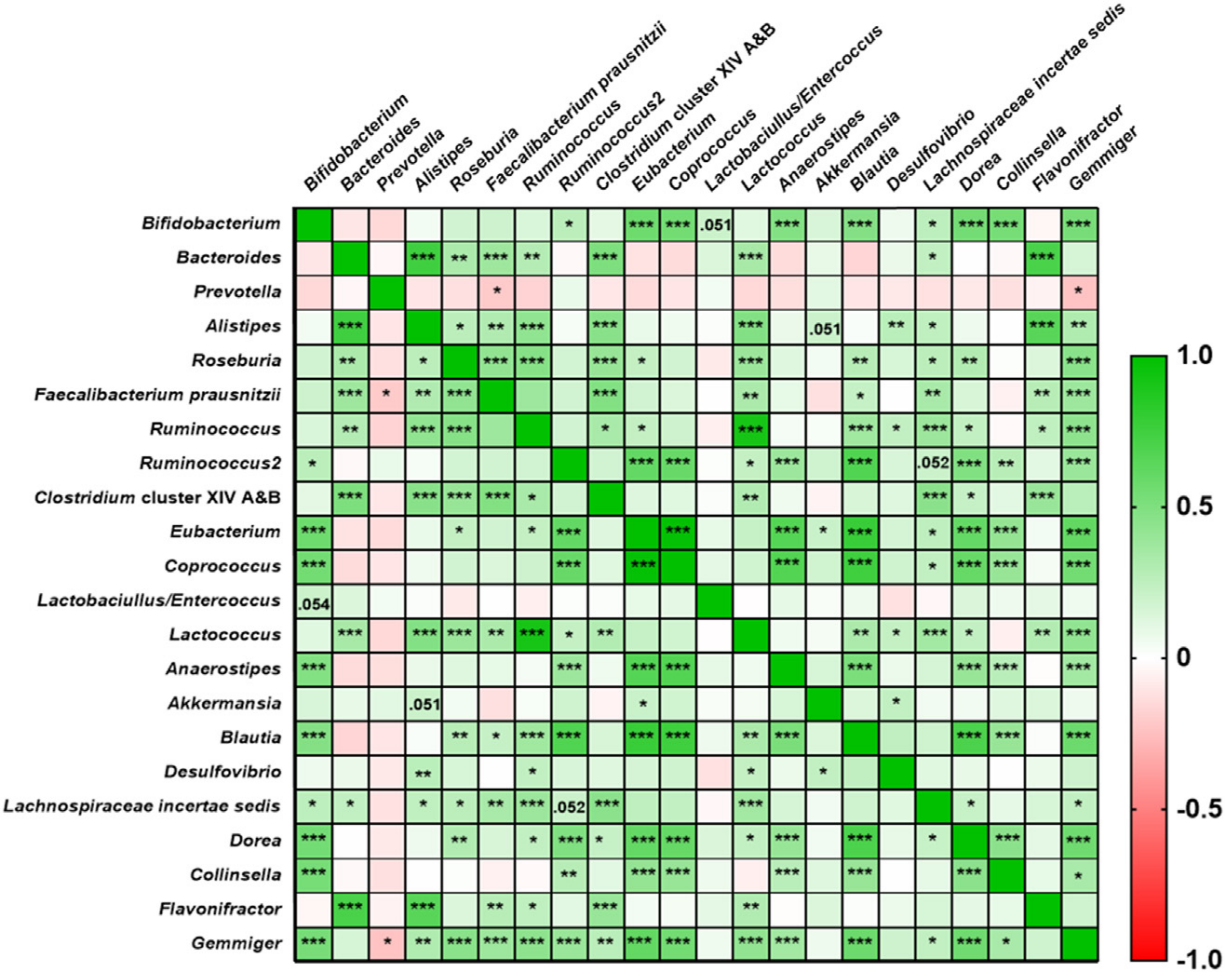
Bacterial taxa-taxa interactions from the entire cohort. Pairwise correlations between bacterial taxa fold change data were calculated using a Spearman’s rank correlation (2-sided adjusted using FDR). Taxa-taxa correlations ranged from -1 to 1 (negative to positive). The depth of the color represents the strength of the correlation. Adjusted *P* (*Q*) values represent significance at ∗ *P* ≤ 0.05, ∗∗ *P* ≤ 0.01 and ∗∗∗ *P* ≤ 0.001.

**FIGURE 14:**
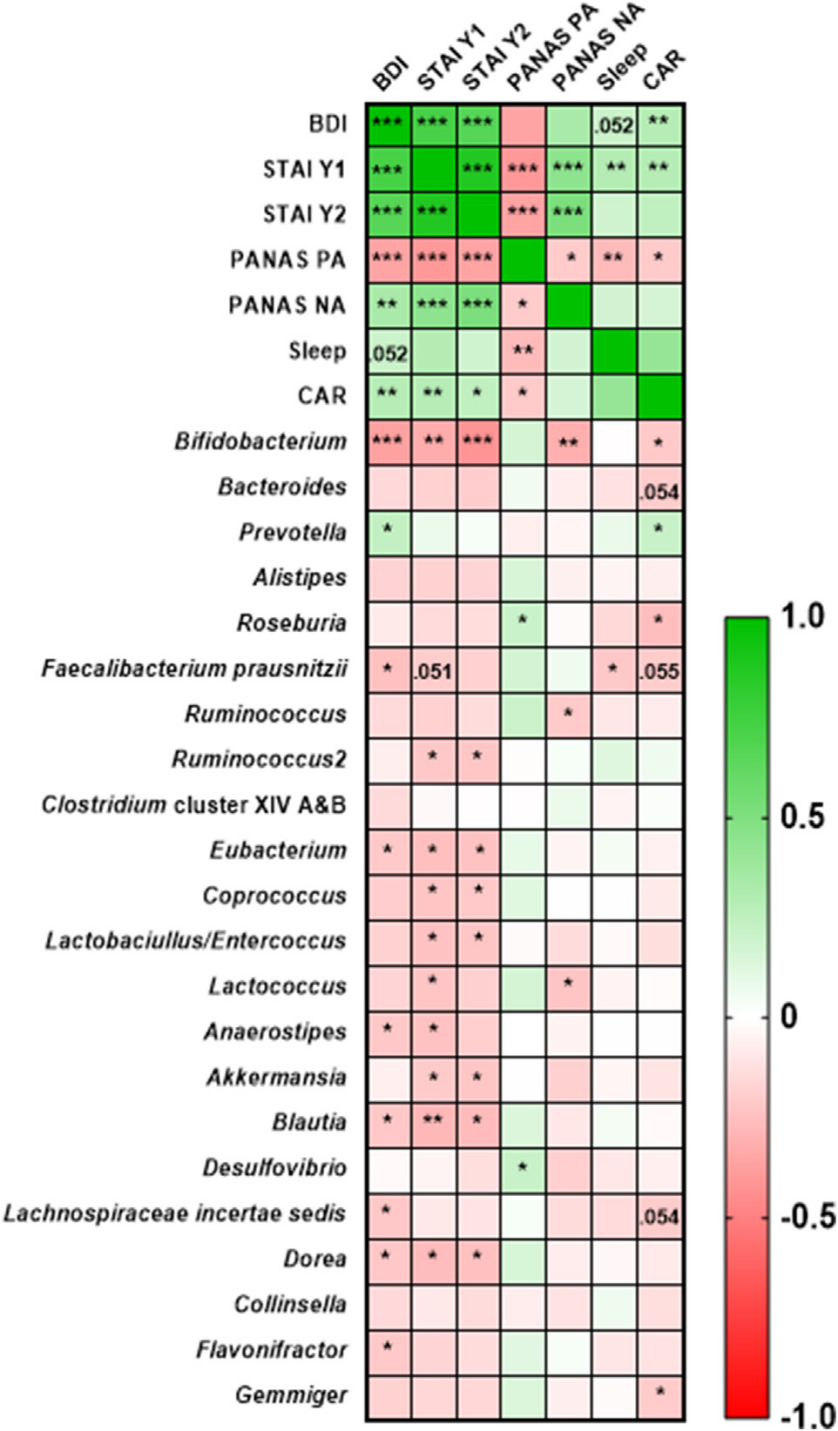
Bacterial taxa-mood state interactions from the entire cohort. Pairwise correlations between bacterial taxa and mood state fold change data were calculated using a Spearman’s rank correlation (2-sided adjusted using FDR). Taxa-mood state correlations ranged from -1 to 1 (negative to positive). The depth of the color represents the strength of the correlation. Adjusted *P* (*Q*) values represent significance at ∗ *P* ≤ 0.05, ∗∗ *P* ≤ 0.01, and ∗∗∗ *P* ≤ 0.001. Abbreviations: BDI, Beck Depression Inventory; CAR, cortisol awakening response; NA, negative affect; PA, positive affect; PANAS, Positive and Negative Affect Schedule; PSQI = Pittsburgh Sleep Quality Index; STAI Y1 and Y2, State Trait Anxiety Inventory;

In order to investigate the relationships between changes in gut microbiota taxa and mood state we constructed a correlation matrix using the fold change ((post-pre)/pre)) for the entire cohort of the QMP bacterial taxa and mood state data. The data were then analyzed using a nonparametric Spearman’s rank correlation corrected using FDR (Figure 13, Figure 14, and Supplemental Table 5).

We observed several significant correlations between both taxa-taxa and taxa-mood state. Regarding taxa-taxa interactions, *Bifidobacterium* was found to be positively correlated with *Eubacterium* (Spearman’s ρ = 0.57, *P* = 1.13 × 10^-7^), *Coprococcus* (Spearman’s ρ = 0.56, *P* = 4.03 × 10^-9^), *Anaerostipes* (Spearman’s ρ = 0.48, *P* = 9.43 × 10^-7^), *Blautia* (Spearman’s ρ = 0.48, *P* = 1.59 × 10^-6^), *Dorea* (Spearman’s ρ = 0.54, *P* = 2.34 × 10^-8^), *Collinsella* (Spearman’s ρ = 0.53, *P* = 4.70 × 10^-8^), *Gemmiger* (Spearman’s ρ = 0.53, *P* = 6.00 × 10^-8^), and to a lesser extent *Lachnospiraceae incertae sedis* (Spearman’s ρ = 0.26, *P* = 0.01), whereas *Bacteroides* were found to be positively correlated with *Alistipes* (Spearman’s ρ = 0.75, *P* = 1.41 × 10^-17^), *Roseburia* (Spearman’s ρ = 0.32, *P* = 0.002), *Faecalibacterium prausnitzii* (Spearman’s ρ = 0.38, *P* = 0.0002), and *Flavonifractor* (Spearman’s ρ = 0.71, *P* = 3.28 × 10^-15^). Interestingly there were also significant correlations found between *Eubacterium* and *Coprococcus* (Spearman’s ρ = 0.98, *P* = 1.3 x 10^-64^) (Figure 13 and Supplemental Table 5).

Regarding taxa-mood state interactions, significant negative correlations were found between *Bifidobacterium* and BDI (Spearman’s ρ = −0.37, *P* = 2.91 × 10^-4^), STAI Y1 (Spearman’s ρ = −0.33, *P* = 0.001), STAI Y2 (Spearman’s ρ = −0.42, *P* = 3.12 × 10^-5^), PANAS-SF NA (Spearman’s ρ = −0.32, *P* = 0.03) as well as CAR (Spearman’s ρ = −0.22, *P* = 0.04). There were also mild correlations found between *Faecalibacterium prausnitzii,* (Spearman’s ρ = −0.20, *P* = 0.02), *Eubacterium* (Spearman’s ρ = −0.21, *P* = 0.041), *Anaerostipes* (Spearman’s ρ = −0.21, *P* = 0.04), *Blautia* (Spearman’s *p* = −0.22, *P* = 0.03), *Lachnospiraceae incertae sedis* (Spearman’s ρ = −0.22, *P* = 0.04), *Dorea* (Spearman’s ρ = −0.22, *P* = 0.04), *Flavonifractor* (Spearman’s ρ = −0.21, *P* = 0.04) and BDI. Additionally, several significant correlations were found between *Eubacterium* (Spearman’s ρ = −0.25, *P* = 0.01), *Coprococcus* (Spearman’s ρ = −0.23, *P* = 0.03), *Lactobacillus/Enterococcus* (Spearman’s ρ = −0.24, *P* = 0.03), *Anaerostipes* (Spearman’s ρ = −0.22, *P* = 0.02), *Akkermansia* (Spearman’s ρ = −0.21, *P* = 0.046) *Blautia* (Spearman’s ρ = −0.27, *P* = 0.007)*, Ruminococcus2* (Spearman’s ρ = −0.22, P = 0.04), and *Dorea* (Spearman’s ρ = −0.26, *P* = 0.01) with STAI Y1, as well as *Eubacterium* (Spearman’s ρ = −0.24, *P* = 0.02), *Coprococcus* (Spearman’s ρ = −0.21, *P* = 0.04), *Lactobacillus/Enterococcus* (Spearman’s ρ = −0.22, *P* = 0.03), *Blautia* (Spearman’s ρ = −0.27, *P* = 0.01)*, Ruminococcus2* (Spearman’s ρ = −0.22, *P* = 0.03), *Akkermansia* (Spearman’s ρ = −0.22, *P* = 0.03), and *Dorea* (Spearman’s ρ = −0.25, *P* = 0.02) with STAI Y2 (Figure 14 and Supplemental Table 5). Furthermore, *Roseburia* was correlated with improvements in PANAS PA scores (Spearman’s ρ = −0.21, *P* = 0.046) and CAR (Spearman’s ρ = −0.26, *P* = 0.01). Increases in *Lactococcus* were also correlated with improvements in PANAS NA scores (Spearman’s ρ = −0.23, *P* = 0.03), whereas *Faecalibacterium prausnitzii* was correlated with improvements in PSQI scores (Spearman’s ρ = −0.22, *P* = 0.04) and *Gemmiger* with improvements in CAR (Spearman’s ρ = −0.21, *P* = 0.049). Finally, increases in *Prevotella* were correlated with worsening BDI scores (Spearman’s ρ = −0.24, *P* = 0.02) and CAR values (Spearman’s ρ = −0.21, *P* = 0.045),

### ^1^H-NMR spectroscopic profiles

**FIGURE 15:**
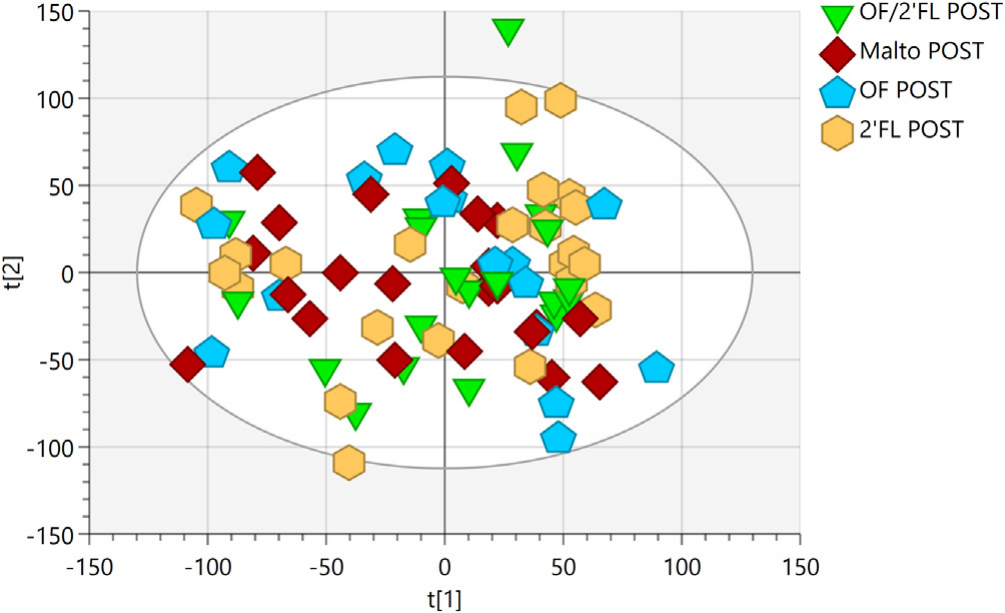
Urinary ^1^H nuclear magnetic resonance (^1^H-NMR) profiles for the entire cohort at completion segregated by intervention. Unsupervised principal component analysis (PCA) scores plot of pre and post intervention urine samples. R^2^Cum = 0.695, Q^2^Cum = 0.431.

Metabolic profiles of urine samples across the 4 interventions were analyzed using unsupervised (PCA) methods to assess whether there were any intrinsic differences between samples collected pre and post intervention (PCA model: 7 principal components, R^2^Cum = 0.695, Q^2^Cum = 0.431) (Figure 15). This analysis revealed no clear clustering of interventions, yet, both OF and combination of OF/2’FL showed trends toward clustering on completion of the intervention (OF R^2^Cum = 0.614, Q^2^Cum = 0.279 and OF/2’FL R^2^Cum = 0.623, Q^2^Cum = 0.217). However, upon performing supervised modeling using OPLS-DA (1 predictive component and 2 orthogonal components), the model was unable to differentiate based on assigned pre and post classifications with predictive ability (OF R^2^Y = 0.778, Q^2^Cum = −0.306 and OF/2’FL R2Y = 0.676, Q^2^Cum = −0.312). Subsequently, no further analysis was carried out.

## Discussion

In this trial we explored the effects of the prebiotic OF and prebiotic candidate 2’FL, alone and in combination, on microbial composition, mood state (BDI, STAI Y1, STAI Y2, PANAS-SF), sleep quality, and CAR in healthy adults with mild-to-moderate levels of anxiety and depression. This is the first study to demonstrate that intake of OF and 2’FL can result in noticeable differences in microbial modulation and substantial improvements in mood, as reflected in BDI, STAI Y1, STAI Y2, PANAS-SF scores, and CAR in adults with mild-to-moderate levels of anxiety and depression.

Large differences in microbial responses were seen between the 4 different interventions, with OF displaying the largest increases in microbial load in a number of different bacterial groups including *Bifidobacterium*, *Bacteroides*, and *Faecalibacterium prausnitzii*. These results coincide with several previous human intervention studies [4,50,51] further confirming the evidence of the high level of selectivity of OF toward *Bifidobacterium* and the wider microbiota [30]. Yet, while combining OF with 2’FL did not induce any complementary effects compared to OF supplementation, OF did offset the lack of changes seen upon consumption of 2’FL in several bacteria including *Bifidobacterium*, *Bacteroides*, *Roseburia* and *Faecalibacterium prausnitzii*.

It is well documented that large interindividual differences in responses exist in terms of bifidobacterial response to HMO supplementation, as the majority of adults do not possess the necessary bifidobacteria required to enzymatically degrade and utilize HMOs [52]. Within our 2’FL cohort, larger increases in *Roseburia*, *Faecalibacterium prausnitzii*,and more interestingly, *Blautia* in comparison to *Bifidobacterium* were documented in several volunteers. This further suggests that a strong relationship exists between an individual’s gut microbiota and microbial responses to HMOs.

One could speculate that increases in *Blautia, Rosburia*, and *Faecalibacterium prausnitzii* might have occurred due to utilization of 2’FL by *Bifidobacterium*. It was recently reported that *Blautia* can grow on the fucose liberated by fucosidase-producing bacteria [53]. Increases in both *Roseburia* and *Faecalibacterium prausnitzii* often occur in the presence of bifidobacteria as a result of cross-feeding on acetate and lactate [54,55]. Furthermore, increases in *Blautia*, *Roseburia*, and *Faecalibacterium prausnitzii* may have occurred as result of 2’FL degradation by *Akkermansia muciniphilia*. *Akkermansia muciniphilia* is considered a keystone species for its role in mucin degradation [56]. As HMOs share large structural similarities with mucin, increases in *Blautia*, *Roseburia*, and *Faecalibacterium prausnitzii* may have also occurred as a result of proliferation on HMO degradation products in the presence of *Akkermansia muciniphilia*. It was recently documented in pure and coculture experiments that *Roseburia* spp. showed little-to-no signs of growth on HMOs, except in the presence of *Akkermansia muciniphilia* [57].

As previously stated, the majority of studies on the effects of prebiotics on cognitive function, mood state, and sleep quality failed to analyze changes in the gut microbiota [[23], [24], [25]]. Although we acknowledge it is hard to establish the exact mechanisms by which the gut microbiota influences mood state, we observed several significant correlations between bacterial taxa, namely *Bifidobacterium*, *Roseburia*, *Anaerostipes*, *Blautia*, and *Faecalibacterium prausnitzii*, and improvements in anxiety, depression, PA and NA scores, PSQI, and CAR values (FIGURE 13, FIGURE 14). On this basis, one could hypothesize that targeted manipulation of the gut microbiota could have a profound effect on mood state and sleep quality via regulations of neurological, immunological, or endocrine pathways [16,19].

Several strains of bifidobacteria, *Lactobacillus* and *Blautia*, are prominent GABA producers [58,59], and these genera significantly increased on either OF, 2’FL, or combination of OF/2’FL (FIGURE 2, FIGURE 3, FIGURE 4 and Supplemental Table 2). Additionally, higher abundances of *Lachnospiraceae* and *Ruminococcus* have been associated with lower levels of major depressive disorder [60], and these taxa were significantly increased upon consumption of OF (*Lachnospiraceae* [*P* = 0.004] and *Ruminococcus* [*P* ≤ 0.001]) (Supplemental Table 2). Furthermore, higher levels of *Faecalibacterium prausnitzii* have been associated with improved sleep quality and lower levels of generalized anxiety [61], and these were also significantly increased upon consumption of OF, OF/2’FL, and 2’FL in our cohort.

We did not measure changes in SCFAs because fecal samples do not give an accurate measure of metabolite production within the colon, and we were unable to collect blood samples as a result of COVID-19 restrictions. The increases in several SCFA-producing bacteria (*Bifidobacterium*, *Roseburia*, *Lachnospiraceae*, and *Faecalibacterium prausnitzii*) on OF, OF/2’FL, and 2’FL likely led to increases in SCFA production. Increased SCFA production may have beneficial effects on mood state as SCFAs play vital roles in the regulation of neurotransmitter production and reduction in inflammatory responses via modulation of anti- and proinflammatory cytokines (IL-6, TNF-α, and IL-1β) and can act as endocrine signaling molecules [19,20]. Additionally, propionate can protect against lipopolysaccharide-mediated blood brain barrier disruption [62], whereas butyrate has been associated with decreased histone acetylation [21]. Furthermore, lower levels of SCFAs have been detected in depressed individuals and nonhuman primate models, whereas higher SCFA concentrations were associated with improved BDI scores [63,64]. Moreover, in male adults, consumption of either low-dose SCFAs (87.1 mmol acetate, 6.6 mmol propionate, and 26.2 mmol butyrate) or high-dose SCFAs (174.2 mmol acetate, 13.3 mmol propionate, and 52.4 mmol butyrate) compared with placebo resulted in significant reductions in salivary cortisol response [65]. It has also been suggested that SCFAs play a role in regulation of circadian rhythm [66].

CAR values were significantly reduced across the OF, 2’FL, and OF/2’FL interventions, with OF showing the largest decreases. Our results are in accordance with those documented in probiotic interventions in healthy adults [67] and anxious students [68], but not those documented by Schmidt et al. [24], who recorded that β-GOS, but not OF, reduced CAR response. Discrepancies in findings are likely due to differences in sample sizes, length of intervention, and supplement dosages. As CAR is often thought to reflect the HPA, CAR values often correlate with levels of stress, anxiety, and sleep quality [66]. Reductions in PSQI scores were also detected across our cohort and reflected those documented in CAR, with OF again displaying the highest reductions. As higher CAR and poor sleep quality can have significant negative impacts on anxiety, depression, and NA and PA scores [69] and are influential markers of health status [70], targeted manipulation of the gut microbiota using OF and/or 2’FL may provide novel approaches to reduce CAR, improving sleep quality (PSQI) and overall mood.

In order to assess changes in stool frequency and stool consistency, the validated Bristol Stool Form Scale was used. In our cohort, significant increases in stool consistency were detected in the OF (both *P* = 0.006) and OF/2’FL (both *P* = 0.007) interventions. Both these results are significantly different compared with the 2’FL intervention (both *P* = 0.001). The softening effect of OF on stool consistency has previously been documented in both healthy and constipated adults [71,72]. Furthermore, in our cohort, significant increases in stool frequency were only documented upon consumption of the OF/2’FL combination (*P* ≤ 0.001). This result is also significantly different to both the maltodextrin (*P* = 0.016) and 2’FL interventions (*P* = 0.014). These results are unsurprising given the high daily stool frequency at baseline seen in our cohort, as changes in bowel frequency upon inulin consumption are often seen in individuals who are constipated or possess lower stool frequency [29,71,73,74]. The mechanisms by which improvements in stool consistency and frequency occurred are probably a result of increases in bacterial mass, combined with the effects of SCFAs on gut motor hormones and the osmotic properties of SCFAs drawing water into the digestive tract, softening stools and thereby making them easier to pass [71].

Similar changes in gastrointestinal sensations including flatulence, intestinal bloating, abdominal pressure, pain, and feeling of fullness were detected across the 4 interventions. Significant increases in flatulence and intestinal bloating upon consumption of OF have been documented previously [7,75,76]. These end values were not, however, significantly different compared with either placebo or the OF/2’FL or 2’FL interventions (all *P* ≥ 0.05), and the slight increases in flatulence or intestinal bloating did not result in any reported discomfort or discontinuation of the study by any of the volunteers and were rated as mild at most.

Finally, during the trial, volunteers were asked not to alter either their dietary intake or lifestyle, and dietary fiber intakes were estimated at 19.66 g/d at completion. These results are in line with the current UK average of 14.9 to 18 g/d and are significantly lower than the current UK recommendation of 30 g/d [77,78]. As dietary analysis revealed no significant changes in dietary fiber intakes between the start and end of the intervention phase, dietary fiber intakes within both the OF and OF/2’FL groups likely increased by 8 g/d from supplementation. This suggests that OF can contribute toward beneficial increases in dietary fiber without significant increases in adverse gastrointestinal reactions occurring but potentially offering benefits for digestive function and mood.

We must acknowledge that one of the limitations of this study is that we chose not to measure SCFAs in feces as feces are not a good proxy for fermentation within the colon. It is still a commonly used reflective measure of levels of fermentation and could have potentially provided us with greater insights into differences in microbial responses between interventions and effects on microbial composition and mood state. Secondly, while care was taken in correcting for multiple comparisons, the testing of multiple secondary outcomes can result in the increased risk of generating and accepting type 1 errors. Lastly, while we found several significant correlations between bacterial taxa and improvements in mood state, we must acknowledge that correlation does not always equal causation, and due care must be taken in the interpretation of these findings.

In conclusion, this is the first study to demonstrate that consumption of the prebiotic OF and prebiotic candidate 2’FL alone and in combination can result in substantial improvements in mood as reflected in BDI, STAI Y1 and Y2, and PANAS-SF scores and CAR. We can also conclude that OF induced substantial changes in microbial composition, especially increasing numbers of *Bifidobacterium*, *Roseburia, Faecalibacterium*, and *Prevotella*. The changes recorded in bacterial taxa correlated with those seen in several mood state parameters. In contrast, supplementation with 2’FL was unable to match OF in terms of changes in microbial composition due to the large heterogeneity seen between individuals. Future studies are needed to identify individual microbial responses to 2’FL supplementation.

## Acknowledgments

We thank Dr Lynne Bell for her help with statistical analysis.

## Author contributions

The authors’ responsibilities were as follows—PPJJ, RAR, CW, AW, ST, JVH: designed research; PJ: conducted research; PJ: analyzed data; PJ: wrote the paper; RAR, JVH, CW: provided feedback on the manuscript; PJ, RAR, AW, CW: had primary responsibility for final content; and all authors: read and approved the final manuscript.

## Conflicts of interest

ST and JVH are employees of Beneo. PPJJ was funded by a PhD studentship by Beneo. RAR and AW supervised PPJJ. RAR has previously supervised PhD students funded by Beneo.

## Funding

This work was supported by the BENEO-Institute - BENEO GmbH Obrigheim (Germany). ST and JVH played a role in study design. JVH provided feedback on manuscript. ST and JVH played no role in decision to publish.

## Data availability

Data described in this manuscript will be available from the corresponding author and the Research Data Service Team upon successful application of the REED data access request (https://redcap.link/data-request).

## Appendix A Supplementary data

Multimedia component1 Multimedia component1
