# Corrigendum to “Inulin-type fructans and 2’fucosyllactose alter both microbial composition and appear to alleviate stress-induced mood state in a working population compared to placebo (maltodextrin): the EFFICAD Trial, a randomized, controlled trial” [Am J Clin Nutr 118(5) 2023 938-955]

**DOI:** 10.1016/j.ajcnut.2024.03.011

**Published:** 2024-03-19

**Authors:** Peter PJ Jackson, Anisha Wijeyesekera, Claire M Williams, Stephan Theis, Jessica van Harsselaar, Robert A Rastall

**Affiliations:** 1 Department of Food and Nutritional Sciences, University of Reading, Reading, United Kingdom; 2 University of Reading, School of Psychology and Clinical Language Science, Reading, United Kingdom; 3 BENEO-Institute, BENEO GmbH, Obrigheim, Germany

The Author regrets that the data availability statement published in the above article was incorrect. The statement should have read as below:

Data supporting the findings reported in this article are held in the University of Reading Research Data Archive at https://doi.org/10.17864/1947.000509. These data are the property of Beneo GmbH and are commercially confidential. Access to the data may be granted for bona fide research purposes subject to a data access agreement. Please refer to the dataset record for further information about the dataset and access conditions.

