# Supplementary material for: Inulin-type fructans and 2’fucosyllactose alter both microbial composition and appear to alleviate stress-induced mood state in a working population compared to placebo (maltodextrin): the EFFICAD Trial, a randomized, controlled trial
Source: Am J Clin Nutr. 2023 Aug 30;118(5):938–55. doi: 10.1016/j.ajcnut.2023.08.016 (PMC10636234; doi:10.1016/j.ajcnut.2023.08.016)
Supplement: Multimedia component1 [file mmc1.docx]

## **Supplementary Table 1 Mean bacterial populations measured using FISH-FLOW at D0 and D28 of the intervention phase**

| **Intervention** | | | | | | | | | | | | | ***P* (b)** |
| --- | --- | --- | --- | --- | --- | --- | --- | --- | --- | --- | --- | --- | --- |
|  | **OF (*n* = 23)** | | | **Maltodextrin (*n* = 23)** | | | **OF/2'FL (*n* = 23)** | | | **2'FL (*n* = 23)** | | |  |
| **Probe** | **D0** | **D28** | ***P* (a)** | **D0** | **D28** | ***P* (a)** | **D0** | **D28** | ***P* (a)** | **D0** | **D28** | ***P* (a)** |  |
| **Eub I-II-III** | 10.00 (0.07) | 10.35 (0.07) | **≤ 0.001** | 9.96 (0.06) | 9.97 (0.07) | 0.972 | 10.03 (0.06) | 10.25 (0.06) | **≤ 0.001** | 10.01 (0.06) | 10.20 (0.06) | **≤ 0.001** | **≤ 0.001** |
| **Bif164** | 8.57 (0.13) | 9.52 (0.12) | **≤ 0.001** | 8.55 (0.13) | 8.37 (0.15) | 0.146 | 8.69 (0.10) | 9.41 (0.13) | **≤ 0.001** | 8.63 (0.14) | 8.93 (0.12) | **0.016** | **≤ 0.001** |

Bacterial groups measured by FISH-FLOW (Log10 cells/mL) using probes: total bacteria (Eub338 I-II-III) and Bifidobacterium (Bif164) collected at D0 and D28 of the intervention phase. Mean and Standard error (SE). **(a)** Significant differences compared with respective D0 and D28 samples are identified with specified *P* values (grey columns) and are result of a general linear model. **(b)** Significant differences between interventions at D28 are indicted by specified *P* values (orange column) and are the result of an ANCOVA employing D28 values as the dependant variable, intervention as a fixed factor and D0 values, sex and PHQ-9 and GAD-7 scores as covariates. **Keywords:** OF = oligofructose; 2’FL = 2’fucosyllactose

## **Supplementary Table 2 Quantitative Microbiome Profiling (QMP) 16S rRNA data measured at D0 and D28 of the intervention phase**

| **Intervention** | | | | | | | | | | | | | |
| --- | --- | --- | --- | --- | --- | --- | --- | --- | --- | --- | --- | --- | --- |
| **Phylum** | **OF (*n* = 23)** | | | **Maltodextrin (*n* = 23)** | | | **OF/2'FL (*n* = 23)** | | | **2'FL (*n* = 23)** | | | ***P* (b)** |
|  | **D0** | **D28** | ***P* (a)** | **D0** | **D28** | ***P* (a)** | **D0** | **D28** | ***P* (a)** | **D0** | **D28** | ***P* (a)** |  |
| ***Actinomycetota (Actinobacteria)*** | 1.43 x 10^9^ (4.06 x 10^8^) | 4.87 x 10^9^ (9.76 x 10^8^) | **≤ 0.001** | 9.18 x 10^9^ (1.65 x 10^8^) | 9.45 x 10^9^ (2.73 x 10^8^) | 0.936 | 9.57 x 10^8^ (1.76 x 10^8^) | 4.41 x 10^9^ (1.24 x 10^9^) | **≤ 0.001** | 1.25 x 10^8^ (2.56 x 10^8^) | 2.10 x 10^9^ (5.51 x 10^8^) | 0.308 | **0.005** |
| ***Bacteroidota (Bacteroidetes)*** | 2.32 x 10^9^ (4.93 x 10^8^) | 5.64 x 10^9^ (1.18 x 10^9^) | **≤ 0.001** | 2.32 x 10^9^ (5.75 x 10^8^) | 1.88 x 10^9^ (3.85 x 10^8^) | 0.675 | 2.88 x 10^9^ (6.36 x 10^8^) | 4.22 x 10^9^ (7.93 x 10^8^) | 0.101 | 2.35 x 10^9^ (4.7 x 10^8^) | 3.30 x 10^9^ (7.40 x 10^8^) | 0.246 | **0.012** |
| ***Pseudomonadota (Proteobacteria)*** | 8.49 x 10^7^ (3.56 x 10^7^) | 1.80 x 10^8^ (6.65x 10^7^) | 0.128 | 9.86 x 10^7^ (3.12 x 10^7^) | 1.64 x 10^8^ (6.59 x 10^7^) | 0.296 | 1.87 x 10^8^ (8.73 x 10^7^) | 1.59 x 10^8^ (6.14 x 10^7^) | 0.651 | 2.96 x 10^7^ (6.09 x 10^6^) | 1.00 x 10^8^ (2.92 x 10^7^ | 0.260 | 0.792 |
| ***Verrucomicrobiota (Verrucomicrobia)*** | 1.06 x 10^8^ (3.11 x 10^7^) | 3.61 x 10^8^ (1.72 x 10^8^) | 0.190 | 1.82 x 10^8^ (7.34 x 10^7^) | 1.20 x 10^8^ (4.81 x 10^7^) | 0.231 | 8.35 x 10^7^ (3.09 x 10^7^) | 1.72 x 10^8^ (9.93 x 10^7^) | 0.268 | 1.30 x 10^8^ (4.66 x 10^7^) | 1.94 x 10^8^ (6.38 x 10^7^) | 0.939 | 0.238 |
| ***Euryarchaeota*** | 9.79 x 10^7^ (5.66 x 10^7^) | 1.65 x 10^8^ (6.36 x 10^7^) | 0.092 | 8.23 x 10^7^ (3.13 x 10^7^) | 5.99 x 10^7^ (2.56 x 10^7^) | 0.572 | 4.12 x 10^7^ (1.85 x 10^7^) | 5.21 x 10^7^ (2.98 x 10^7^) | 0.783 | 8.51 x 10^7^ (6.51 x 10^7^) | 1.90 x 10^7^ (1.04 x 10^7^) | 0.096 | **0.019** |
| ***Mycoplasmatota (Tenericutes)*** | 5.24 x 10^8^ (1.71 x 10^8^) | 5.60 x 10^8^ (1.25 x 108) | 0.734 | 6.49 x 10^8^ (2.41 x 10^8^) | 6.46 x 10^8^ (2.81 x 10^8^) | 0.979 | 4.57 x 10^8^ (1.44 x 10^8^) | 4.22 x 10^8^ (1.50 x 10^8^) | 0.746 | 3.24 x 10^8^ (7.36 x 10^7^) | 3.81 x 10^8^ (9.93 x 10^7^) | 0.595 | 0.943 |

Quantitative Microbiome Profiling 16S rRNA sequencing data (phylum level) from samples collected at D0 and D28 of the intervention phase. Mean and Standard error (SE). **(a)** Significant differences compared with respective D0 and D28 samples are identified with specified *P* values (grey columns) and are result of a general linear model. **(b)** Significant differences between interventions at D28 are indicted by specified *P* values (orange column) and are the result of an ANCOVA employing D28 values as the dependant variable, intervention as a fixed factor and D0 values, sex and PHQ-9 and GAD-7 scores as covariates. **Keywords:** OF = oligofructose; 2’FL = 2’fucosyllactose

| **Intervention** | | | | | | | | | | | | | |
| --- | --- | --- | --- | --- | --- | --- | --- | --- | --- | --- | --- | --- | --- |
| **Phylum** | **OF (*n* = 23)** | | | **Maltodextrin (*n* = 23)** | | | **OF/2'FL (*n* = 23)** | | | **2'FL (*n* = 23)** | | | ***P* (b)** |
|  | **D0** | **D28** | ***P* (a)** | **D0** | **D28** | ***P* (a)** | **D0** | **D28** | ***P* (a)** | **D0** | **D28** | ***P* (a)** |  |
| ***Fusobacteria*** | 9.37 x 10^5^  (9.37 x 10^5^) | 0 | 0.994 | 0 | 0 | 1.00 | 4.03 x 10^5^ (4.03 x 10^5^) | 2.59 x 10^7^ (2.59 x 10^7^) | **0.049** | 4.03 x 10^5^ (4.03 x 10^5^) | 2.09 x 10^5^ (2.09 x 10^5^) | 0.988 | 0.316 |
| ***Bacillota (firmicutes)*** | 8.48 x 10^9^ (1.36 x 10^9^) | 1.61 x 10^10^ (2.11 x 10^9^) | **≤ 0.001** | 6.84 x 10^9^ (1.07 x 10^9^) | 8.35 x 10^9^ (1.23 x 10^9^) | 0.386 | 8.47 x 10^9^ (1.21 x 10^9^) | 1.23 x 10^10^ (1.91 x 10^9^) | **0.008** | 8.36 x 10^9^ (9.86 x 10^8^) | 1.25 x 10^10^ (1.55 x 10^9^) | **0.004** | **0.021** |

Quantitative Microbiome Profiling 16S rRNA sequencing data (phylum level) from samples collected at D0 and D28 of the intervention phase. Mean and Standard error (SE). **(a)** Significant differences compared with respective D0 and D28 samples are identified with specified *P* values (grey columns) and are results of a general linear model. **(b)** Significant differences between interventions at D28 are indicted by specified *P* values (orange column) and are the result of an ANCOVA employing D28 values as the dependant variable, intervention as a fixed factor and D0 values, sex and PHQ-9 and GAD-7 scores as covariates. **Keywords:** OF = oligofructose; 2’FL = 2’fucosyllactose

| **Intervention** | | | | | | | | | | | | | ***P* (b)** |
| --- | --- | --- | --- | --- | --- | --- | --- | --- | --- | --- | --- | --- | --- |
| **Genus** | **OF (*n* = 23)** | | | **Maltodextrin (*n* = 23)** | | | **OF/2’FL (*n* = 23)** | | | **2’FL (*n* = 23)** | | |  |
|  | **D0** | **D28** | ***P* (a)** | **D0** | **D28** | ***P* (a)** | **D0** | **D28** | ***P* (a)** | **D0** | **D28** | ***P* (a)** |  |
| ***Bifidobacterium*** | 1.16 x 10^9^ (3.75 x 10^8^) | 4.28 x 10^9^ (9.03 x 10^8^) | **≤ 0.001** | 6.96 x 10^8^ (1.67 x 10^8^) | 7.69 x 10^8^ (2.55 x 10^8^) | 0.898 | 8.18 x 10^8^ (1.62 x 10^8^) | 4.05 x 10^9^ (1.23 x 10^9^) | **≤ 0.001** | 9.41 x 10^8^ (2.19 x 10^8^) | 1.76 x 10^9^ (5.33 x 10^8^) | 0.314 | **0.010** |
| ***Bacteroides*** | 1.68 x 10^9^ (3.98 x 10^8^) | 4.21 x 10^9^ (9.41 x 10^8^) | **≤ 0.001** | 1.53 x 10^9^ (4.55 x 10^8^) | 1.32 x 10^9^ (3.08 x 10^8^) | 0.705 | 2.22 x 10^9^ (5.54 x 10^8^) | 3.18 x 10^9^ (7.12 x 10^8^) | 0.154 | 1.78 x 10^9^ (4.11 x 10^8^) | 2.23 x 10^9^ (5.75 x 10^8^) | 0.503 | **0.016** |
| ***Prevotella*** | 1.12 x 10^8^ (5.71 x 10^7^) | 5.55 x 10^8^ (2.01 x 10^8^) | **0.013** | 4.24 x 10^8^ (3.43 x 10^8^) | 3.31 x 10^8^ (2.48 x 10^8^) | 0.665 | 1.12 x 10^8^ (6.79 x 10^7^) | 4.78 x 10^8^ 3.37 x 10^8^) | **0.039** | 7.38 x 10^7^ (3.24 x 10^7^) | 2.65 x 10^8^ (1.05 x 10^8^) | 0.278 | 0.253 |
| ***Alistipes*** | 3.45 x 10^8^ (7.61 x 10^7^) | 6.99 x 10^8^ (2.09 x 10^8^) | **0.004** | 2.42 x 10^8^ (6.50 x 10^7^) | 1.46 x 10^8^ (2.99 x 10^7^) | 0.378 | 3.20 x 10^8^ (7.08 x 10^7^) | 3.31 x 10^8^ (8.17 x 10^7^) | 0.926 | 3.05 x 10^8^ (7.03 x 10^7^) | 3.60 x 10^8^ (9.21 x 10^7^) | 0.650 | **0.024** |
| ***Roseburia*** | 9.37 x 10^8^ (2.42 x 10^8^) | 1.74 x 10^9^ (4.01 x 10^8^) | **0.008** | 8.22 x 10^8^ (2.52 x 10^8^) | 1.10 x 10^9^ (2.61 x 10^8^) | 0.332 | 8.55 x 10^8^ (1.67 x 10^8^) | 1.84 x 10^9^ (4.11 x 10^8^) | **0.001** | 6.23 x 10^8^ (1.24 x 10^8^) | 1.30 x 10^9^ (2.92 x 10^8^) | **0.026** | 0.406 |
| ***Faecalibacterium prausnitzii*** | 5.82 x 10^8^ (9.94 x 10^7^) | 1.93 x 10^9^ (4.16 x 10^8^) | **≤ 0.001** | 7.65 x 10^8^ (2.75 x 10^8^) | 9.85 x 10^8^ (2.15 x 10^8^) | 0.444 | 1.12 x 10^9^ (3.30 x 10^8^) | 1.80 x 10^9^ (4.48 x 10^8^) | **0.015** | 6.20 x 10^8^ (1.43 x 10^8^) | 1.20 x 10^9^ (1.76 x 10^8^) | **0.047** | 0.070 |
| ***Ruminococcus*** | 7.73 x 10^8^ (1.52 x 10^8^) | 2.24 x 10^9^ (5.48 x 10^8^) | **≤ 0.001** | 4.76 x 10^8^ (7.72 x 10^7^) | 6.34 x 10^8^ (1.28 x 10^8^) | 0.603 | 8.10 x 10^8^ (1.89 x 10^8^) | 1.21 x 10^9^ (4.14 x 10^8^) | 0.239 | 7.06 x 10^8^ (1.51 x 10^8^) | 1.20 x 10^9^ (3.73 x 10^8^) | 0.142 | 0.059 |

Quantitative Microbiome Profiling 16S rRNA sequencing data (genus level) from samples collected at D0 and D28 of the intervention phase. Mean and Standard error (SE). **(a)** Significant differences compared with respective D0 and D28 samples are identified with specified *P* values (grey columns) and are result of a general linear model. **(b)** Significant differences between interventions at D28 are indicted by specified *P* values (orange column) and are the result of an ANCOVA employing D28 values as the dependant variable, intervention as a fixed factor and D0 values, sex and PHQ-9 and GAD-7 scores as covariates. **Keywords:** OF = oligofructose; 2’FL = 2’fucosyllactose

| **Genus (continued)** | **OF (*n* = 23)** | | | **Maltodextrin (*n* = 23)** | | | **OF/2’FL (*n* = 23)** | | | **2’FL (*n* = 23)** | | | ***P* (b)** |
| --- | --- | --- | --- | --- | --- | --- | --- | --- | --- | --- | --- | --- | --- |
|  | **D0** | **D28** | ***P* (a)** | **D0** | **D28** | ***P* (a)** | **D0** | **D28** | ***P* (a)** | **D0** | **D28** | ***P* (a)** |  |
| ***Ruminococcus2*** | 2.94 x 10^8^ (6.11 x 10^7^) | 4.36 x 10^8^ (1.30 x 10^8^) | 0.155 | 3.11 x 10^8^ (6.50 x 10^7^) | 3.92 x 10^8^ (9.87 x 10^7^) | 0.431 | 3.09 x 10^8^ (7.08 x 10^7^) | 3.90 x 10^8^ (9.92 x 10^7^) | 0.415 | 4.39 x 10^8^ (1.24 x 10^8^) | 6.44 x 10^8^ (1.41 x 10^8^) | **0.040** | 0.653 |
| ***Clostridium Cluster* 14A&B** | 2.27 x 10^8^ (5.40 x 10^7^) | 3.06 x 10^8^ (4.12 x 10^7^) | 0.163 | 1.71 x 10^8^ (3.42 x 10^7^) | 2.48 x 10^8^ (5.23 x 10^7^) | 0.423 | 3.35 x 10^8^ (9.03 x 10^7^) | 2.34 x 10^8^ (4.55 x 10^7^) | 0.073 | 2.65 x 10^8^ (6.55 x 10^7^) | 3.36 x 10^8^ (5.82 x 10^7^) | 0.082 | **0.038** |
| ***Eubacterium*** | 1.17 x 10^8^ (2.20 x 10^7^) | 2.33 x 10^8^ (6.57 x 10^7^) | **0.003** | 8.70 x 10^7^ (1.62 x 10^7^) | 9.75 x 10^7^ (2.01 x 10^7^) | 0.702 | 9.94 x 10^7^ (2.13 x 10^7^) | 1.13 x 10^8^ (2.51 x 10^7^) | 0.714 | 9.73 x 10^7^ (1.87 x 10^7^) | 1.90 x 10^8^ (4.19 x 10^7^) | **0.016** | 0.148 |
| ***Coprococcus*** | 7.02 x 10^8^ (2.75 x 10^8^) | 9.12 x 10^8^ (1.59 x 10^8^) | 0.155 | 3.57 x 10^8^ (5.18 x 10^7^) | 4.64 x 10^8^ (1.16 x 10^8^) | 0.400 | 4.63 x 10^8^ (8.23 x 10^7^) | 4.89 x 10^8^ (9.99 x 10^7^) | 0.863 | 7.09 x 10^8^ (1.47 x 10^8^) | 8.27 x 10^8^ (1.34 x 10^8^) | 0.417 | 0.135 |
| ***Lactobacillus/Enterococcus*** | 9.26 x 10^7^ (6.33 x 10^7^) | 3.08 x 10^8^ (1.78 x 10^8^) | **0.029** | 7.80 x 10^6^ (6.93 x 10^6^) | 3.19 x 10^6^ (2.62 x 10^6^) | 0.964 | 9.55 x 10^7^ (7.00 x 10^7^) | 1.42 x 10^8^ (1.19 x 10^8^) | 0.634 | 2.90 x 10^7^ (1.54 x 10^7^) | 5.24 x 10^7^ (3.19 x 10^7^) | 0.810 | 0.393 |
| ***Lactococcus*** | 6.93 x 10^6^ (4.60 x 10^6^) | 1.13 x 10^6^ (6.73 x 10^5^) | 0.098 | 1.48 x 10^6^ (1.23 x 10^6^) | 1.77 x 10^6^ (8.34 x 10^5^) | 0.926 | 2.36 x 10^6^ (1.17 x 10^6^) | 8.72 x 10^5^ (3.48 x 10^5^) | 0.669 | 6.26 x 10^6^ (4.67 x 10^6^) | 3.09 x 10^6^ (1.29 x 10^6^) | 0.366 | 0.252 |
| ***Anaerostipes*** | 5.50 x 10^8^ (2.86 x 10^8^) | 6.19 x 10^8^ (1.60 x 10^8^) | 0.658 | 1.99 x 10^8^ (3.91 x 10^7^) | 3.42 x 10^8^ (9.34 x 10^7^) | 0.328 | 2.37 x 10^8^ (4.62 x 10^7^) | 2.78 x 10^8^ (6.70 x 10^7^) | 0.791 | 4.34 x 10^8^ (1.26 x 10^8^) | 5.17 x 10^8^ (1.11 x 10^7^) | 0.658 | 0.373 |
| ***Akkermansia*** | 1.06 x 10^8^ (3.11 x 10^7^) | 3.60 x 10^8^ (1.71 x 10^8^) | **0.015** | 1.91 x 10^8^ (7.63 x 10^7^) | 1.20 x 10^8^ (4.81 x 10^7^) | 0.536 | 8.35 x 10^7^ (3.09 x 10^7^) | 1.84 x 10^8^ (9.91 x 10^7^) | 0.330 | 1.31 x 10^8^ (4.66 x 10^7^) | 1.91 x 10^8^ (6.41 x 10^7^) | 0.556 | 0.264 |

Quantitative Microbiome Profiling 16S rRNA sequencing data (genus level) from samples collected at D0 and D28 of the intervention phase. Mean and Standard error (SE). **(a)** Significant differences compared with respective D0 and D28 samples are identified with specified *P* values (grey columns) and are result of a general linear model. **(b)** Significant differences between interventions at D28 are indicted by specified *P* values (orange column) and are the result of an ANCOVA employing D28 values as the dependant variable, intervention as a fixed factor and D0 values, sex and PHQ-9 and GAD-7 scores as covariates. **Keywords:** OF = oligofructose; 2’FL = 2’fucosyllactose

| **Genus (continued)** | **OF (*n* = 23)** | | | **Maltodextrin (*n* = 23)** | | | **OF/2’FL (*n* = 23)** | | | **2’FL (*n* = 23)** | | | ***P* (b)** |
| --- | --- | --- | --- | --- | --- | --- | --- | --- | --- | --- | --- | --- | --- |
|  | **D0** | **D28** | ***P* (a)** | **D0** | **D28** | ***P* (a)** | **D0** | **D28** | ***P* (a)** | **D0** | **D28** | ***P* (a)** |  |
| ***Blautia*** | 1.59 x 10^9^ (3.57 x 10^8^) | 1.83 x 10^9^ (3.25 x 10^8^) | 0.486 | 1.54 x 10^9^ (3.70 x 10^8^) | 1.39 x 10^9^ (4.10 x 10^8^) | 0.691 | 1.31 x 10^9^ (2.41 x 10^8^) | 1.62 x 10^9^ (3.53 x 10^8^) | 0.373 | 1.78 x 10^9^ (2.87 x 10^8^) | 2.42 x 10^9^ (4.72 x 10^8^) | 0.063 | 0.315 |
| ***Desulfovibrio*** | 1.31 x 10^7^ (7.38 x 10^7^) | 4.57 x 10^7^ (1.66 x 10^7^) | **0.021** | 2.02 x 10^7^ (9.94 x 10^6^) | 3.25 x 10^7^ (1.61 x 10^7^) | 0.339 | 3.64 x 10^7^ (3.32 x 10^7^) | 7.02 x 10^7^ (5.46 x 10^7^) | **0.017** | 7.69 x 10^6^ (3.86 x 10^6^) | 1.66 x 10^7^ (9.17 x 106) | 0.523 | 0.155 |
| ***Lachnospiraceae incertae sedis*** | 3.41 x 10^8^ (7.36 x 10^7^) | 6.23 x 10^8^ (1.14 x 10^8^) | **0.004** | 2.81 x 10^8^ (4.47 x 10^7^) | 5.40 x 10^8^ (1.34 x 10^8^) | 0.066 | 4.47 x 10^8^ (9.36 x 10^7^) | 5.45 x 10^8^ (1.03 x 10^8^) | 0.341 | 4.90 x 10^8^ (9.48 x 10^7^) | 6.10 x 10^8^ (1.06 x 10^8^) | 0.243 | 0.586 |
| ***Dorea*** | 2.97 x 10^8^ (5.54 x 10^7^) | 4.67 x 10^8^ (9.64 x 10^7^) | 0.091 | 3.01 x 10^8^ (6.37 x 10^7^) | 2.88 x 10^8^ (7.38 x 10^7^) | 0.993 | 3.44 x 10^8^ (5.87 x 10^7^) | 4.30 x 10^8^ (9.78 x 10^7^) | 0.381 | 5.54 x 10^8^ (1.09 x 10^8^) | 4.75 x 10^8^ (9.67 x 10^7^) | 0.436 | 0.565 |
| ***Collinsella*** | 1.52 x 10^8^ (4.39 x 10^7^) | 2.89 x 10^8^ (6.29 x 10^7^) | **0.007** | 7.69 x 10^7^ (1.87 x 10^7^) | 9.43 x 10^7^ (2.90 x 10^7^) | 0.671 | 7.33 x 10^7^ (1.91 x 10^7^) | 2.14 x 10^8^ (4.68 x 10^7^) | **0.006** | 1.83 x 10^8^ (5.09 x 10^7^) | 2.19 x 10^8^ (5.83 x 10^7^) | 0.470 | 0.140 |
| ***Flavonifractor*** | 2.75 x 10^7^ (7.82 x 10^6^) | 7.82 x 10^7^ (1.58 x 10^7^) | **0.011** | 4.77 x 10^7^ (1.32 x 10^7^) | 5.32 x 10^7^ (1.76 x 10^7^) | 0.659 | 7.09 x 10^7^ (2.00 x 10^7^) | 8.22 x 10^7^ (2.15 x 10^7^) | 0.563 | 5.62 x 10^7^ (1.10 x 10^7^) | 7.52 x 10^7^ (1. 68x 10^7^) | 0.335 | 0.252 |
| ***Gemmiger*** | 4.14 x 10^8^ (1.09 x 10^8^) | 5.21 x 10^8^ (8.79 x 10^7^) | 0.126 | 2.80 x 10^8^ (7.26 x 10^7^) | 2.38 x 10^8^ (6.44 x 10^7^) | 0.662 | 2.81 x 10^8^ (5.97 x 10^7^) | 4.31 x 10^8^ (1.05 x 10^8^) | **0.033** | 2.81 x 10^8^ (5.68 x 10^7^) | 2.83 x 10^8^ (4.45 x 10^7^) | 0.978 | 0.079 |

Quantitative Microbiome Profiling 16S rRNA sequencing data (genus level) from samples collected at D0 and D28 of the intervention phase. Mean and Standard error (SE). **(a)** Significant differences compared with respective D0 and D28 samples are identified with specified *P* values (grey columns) and are results of a general linear model. **(b)** Significant differences between interventions at D28 are indicted by specified *P* values (orange column) and are the result of an ANCOVA employing D28 values as the dependant variable, intervention as a fixed factor and D0 values, sex and PHQ-9 and GAD-7 scores as covariates. **Keywords:** OF = oligofructose; 2’FL = 2’fucosyllactose

## **Supplementary Table 3 Mean bowel habit diary data: Stool consistency, Frequency, Flatulence, Intestinal bloating, Abdominal Pressure, Abdominal Pain and Feeling of Fullness measured at run-in week and last of the intervention phase**

| **Intervention** | | | | | | | | | | | | | ***P* (b)** | |
| --- | --- | --- | --- | --- | --- | --- | --- | --- | --- | --- | --- | --- | --- | --- |
| **Gastrointestinal sensation and bowel habit** | **OF (*n* = 23)** | | | **Maltodextrin (*n* = 23)** | | | **OF/2’FL (*n* = 23)** | | | **2’FL (*n* = 23)** | | |  |  |
|  | **Run-in** | **D22-D28** | ***P* (a)** | **Run-in** | **D22-D28** | ***P* (a)** | **Run-in** | **D22-D28** | ***P* (a)** | **Run-in** | **D22-D28** | ***P* (a)** |  |  |
| **Stool Frequency** | 1.44 (0.12) | 1.45 (0.13) | 0.904 | 1.21 (0.08) | 1.21 (0.08) | 0.952 | 1.45 (0.14) | 1.82 (0.17) | **≤ 0.001** | 1.28 (0.10) | 1.26 (0.09) | 0.810 | **0.006** |  |
| **Stool Consistency** | 3.88 (0.15) | 4.36 (0.15) | **0.006** | 3.83 (0.17) | 4.02 (0.19) | 0.285 | 3.89 (0.19) | 4.35 (0.17) | **0.007** | 3.73 (0.21) | 3.50 (0.17) | 0.168 | **≤ 0.001** |  |
| **Flatulence** | 0.42 (0.08) | 0.73 (0.10) | **0.001** | 0.63 (0.10) | 0.72 (0.10) | 0.353 | 0.68 (0.09) | 0.78 (0.09) | 0.320 | 0.52 (0.08) | 0.55 (0.10) | 0.790 | 0.242 |  |
| **Intestinal Bloating** | 0.32 (0.08) | 0.58 (0.11) | **0.007** | 0.55 (0.12) | 0.57 (0.12) | 0.816 | 0.47 (0.12) | 0.44 (0.12) | 0.739 | 0.34 (0.11) | 0.29 (0.08) | 0.595 | 0.098 |  |
| **Abdominal Pain** | 0.137 (0.04) | 0.21 (0.07) | 0.287 | 0.22 (0.08) | 0.21 (0.05) | 0.771 | 0.20 (0.06) | 0.21 (0.06) | 0.923 | 0.23 (0.06) | 0.14 (0.05) | 0.148 | 0.563 |  |
| **Abdominal Pressure** | 0.21 (0.07) | 0.35 (0.09) | 0.096 | 0.42 (0.10) | 0.40 (0.10) | 0.834 | 0.40 (0.10) | 0.35 (0.011) | 0.576 | 0.29 (0.09) | 0.24 (0.08) | 0.676 | 0.563 |  |
| **Feeling of Fullness** | 0.44 (0.12) | 0.45 (0.12) | 0.916 | 0.63 (0.12) | 0.65 (0.12) | 0.874 | 0.53 (0.13) | 0.53 (0.14) | 1.00 | 0.33 (0.10) | 0.36 (0.10) | 0.772 | 0.830 |  |

Gastrointestinal sensation and bowel habit scores at D0 and D28 of the intervention phase. Mean and Standard error (SE). **(a)** Significant differences between respective run-in phase and last week of the intervention phase (Day 22-28) interventions are identified with specified *P* values (grey columns) and are result of a linear marginal model. **(b)** Significant differences between interventions at D28 are indicted by specified *P* values (orange column) and are the result of an ANCOVA employing D28 scores as the dependant variable, intervention as a fixed factor and D0 values, sex and PHQ-9 and GAD-7 scores as covariates. **Keywords:** OF = oligofructose; 2’FL = 2’fucosyllactose

## **Supplementary Table 4 Mean mood state scores across all four interventions at D0 and D28 of the intervention phase**

| **Intervention** | | | | | | | | | | | |  | ***P* (b)** |
| --- | --- | --- | --- | --- | --- | --- | --- | --- | --- | --- | --- | --- | --- |
| **Mood state** | **OF (*n* = 23)** | | | **Maltodextrin (*n* = 23)** | | | **OF/2’FL (*n* = 23)** | | | **2’FL (*n* = 23)** | | |  |
|  | **D0** | **D28** | ***P* (a)** | **D0** | **D28** | ***P* (a)** | **D0** | **D28** | ***P* (a)** | **D0** | **D28** | ***P* (a)** |  |
| **Becks Depression Inventory** | 18.96 (0.96) | 8.52 (0.92) | **≤ 0.001** | 18.04 (0.89) | 16.04 (1.14) | 0.074 | 19.09 (0.83) | 9.52 (0.92) | **≤ 0.001** | 17.83 (0.75) | 13.13 (1.41) | **≤ 0.001** | **≤ 0.001** |
| **State Trait Anxiety Inventory Y1** | 54.91 (1.11) | 41.61 (1.62) | **≤ 0.001** | 55.91 (1.73) | 52.30 (1.28) | 0.063 | 55.35 (1.10) | 42.04 (1.82) | **≤ 0.001** | 52.91 (1.19) | 46.43 (1.64) | **0.001** | **≤ 0.001** |
| **State Trait Anxiety Inventory Y2** | 55.70 (1.05) | 42.35 (1.85) | **0.002** | 55.17 (1.42) | 52.65 (1.68) | 0.086 | 56.00 (1.145) | 45.26 (1.80) | **≤ 0.001** | 53.52 (0.71) | 49.00 (1.52) | **≤ 0.001** | **≤ 0.001** |
| **Positive Affect** | 26.30 (1.14) | 34.17 (1.29) | **≤ 0.001** | 24.91 (1.22) | 26.78 (1.06) | 0.176 | 24.61 (0.93) | 29.39 (1.20) | **≤ 0.001** | 24.48 (1.26) | 31.91 (1.25) | **≤ 0.001** | **≤ 0.001** |
| **Negative Affect** | 32.09 (1.21) | 23.48 (0.99) | **≤ 0.001** | 28.91 (0.98) | 28.74 (1.24) | 0.878 | 28.65 (1.15) | 23.78 (0.76) | **≤ 0.001** | 29.96 (0.95) | 25.00 (1.02) | **≤ 0.001** | **≤ 0.001** |
| **Pittsburgh Sleep Quality Index** | 6.61 (0.46) | 5.26 (0.45) | **≤ 0.001** | 6.91 (0.45) | 5.83 (0.50) | **0.004** | 6.61 (0.48) | 5.48 (0.47) | **0.003** | 6.00 (0.32) | 4.65 (0.38) | **≤ 0.001** | 0.715 |
| **Cortisol Awakening Response** | 22.31 (0.68) | 20.32 (0.76) | **≤ 0.001** | 21.89 (0.60) | 22.26 (0.69) | 0.300 | 22.82 (0.92) | 20.89 (0.95) | **≤ 0.001** | 22.13 (0.61) | 21.17 (0.70) | **0.008** | **≤ 0.001** |

Mood state scores and cortisol awakening response values at Day 0 and Day 28 of the intervention phase. Mean and Standard error (SE). **(a)** Significant differences within respective D0 and D28 scores/values are identified with specified P values (grey columns) and are result of a linear marginal model. (**b)** Significant differences between interventions at D28 are indicted by specified *P* values (orange column) and are the result of an ANCOVA employing D28 scores/values as the dependant variable, intervention as a fixed factor and D0 values, sex and PHQ-9 and GAD-7 scores as covariates. **Keywords:** OF = oligofructose; 2’FL = 2’fucosyllactose

## **Supplementary Table 5 Bacterial taxa -taxa and taxa– mood state correlation matrix**

**Bacterial Taxa – mood state correlation matrix**

|  | **BDI** | **STAI Y1** | **STAI Y2** | **PANAS PA** | **PANAS NA** | **PSQI** | **CAR** |
| --- | --- | --- | --- | --- | --- | --- | --- |
| **BDI** | 1.00 | 0.72 | 0.66 | -0.36 | 0.336 | 0.203 | 0.283 |
|  | 1.00 | **6.44x10^-16^** | **9.42x10^-13^** | **4.61x10^-04^** | **0.001** | **0.052** | **0.006** |
| **STAI Y1** | 0.72 | 1.00 | 0.86 | -0.40 | 0.440 | 0.290 | 0.282 |
|  | **6.44x10^-16^** | 1.00 | **1.14x10^-28^** | **7.73x10^-05^** | **1.12x10^-05^** | **0.005** | **0.006** |
| **STAI Y2** | 0.66 | 0.86 | 1.00 | -0.36 | 0.514 | 0.187 | 0.243 |
|  | **9.42x10^-13^** | **1.14x10^-28^** | 1.00 | **3.57x10^-04^** | **1.64x10^-07^** | **0.074** | **0.019** |
| **PANAS PA** | -0.36 | -0.40 | -0.36 | 1.00 | -0.205 | -0.273 | -0.208 |
|  | **4.61x10^-04^** | **7.73x10^-05^** | **3.57x10^-04^** | 1.00 | **0.049** | **0.008** | **0.046** |
| **PANAS NA** | 0.34 | 0.440 | 0.514 | -0.21 | 1.00 | 0.18 | 0.16 |
|  | **0.001** | **1.12x10^-05^** | **1.64x10^-07^** | **0.049** | 1.00 | **0.09** | 0.12 |
| **PSQI** | 0.20 | 0.29 | 0.19 | -0.273 | 0.18 | 1.00 | 0.41 |
|  | **0.05** | **0.005** | **0.074** | **0.008** | **0.09** | 1.00 | **5.0x10^-05^** |
| **Car** | 0.28 | 0.28 | 0.24 | -0.21 | 0.16 | 0.41 | 1.00 |
|  | **0.006** | **0.006** | **0.019** | **0.046** | 0.12 | **5.0x10^-05^** | 1.00 |
| ***Bifidobacterium*** | -0.37 | -0.33 | -0.42 | 0.17 | -0.32 | -0.01 | -0.22 |
|  | **2.91x10^-04^** | **0.001** | **3.1x10^-05^** | 0.11 | **0.03** | 0.94 | **0.04** |
| ***Bacteroides*** | -0.15 | -0.18 | -0.20 | 0.05 | -0.073 | -0.121 | -0.201 |
|  | 0.15 | **0.08** | **0.06** | 0.61 | 0.486 | 0.252 | **0.055** |
| ***Prevotella*** | 0.24 | 0.08 | 0.03 | -0.06 | -0.04 | 0.08 | 0.21 |
|  | **0.02** | 0.46 | 0.79 | 0.54 | 0.68 | 0.44 | **0.045** |
| ***Alistipes*** | -0.17 | -0.18 | -0.17 | 0.15 | -0.06 | -0.04 | -0.07 |
|  | 0.10 | **0.09** | 0.10 | 0.15 | 0.58 | 0.68 | 0.52 |
| ***Roseburia*** | -0.09 | -0.14 | -0.13 | 0.21 | -0.02 | -0.15 | -0.26 |
|  | 0.42 | 0.17 | 0.20 | **0.046** | 0.84 | 0.14 | **0.01** |
| ***Faecalibacterium prausnitzii*** | -0.25 | -0.20 | -0.18 | 0.17 | 0.06 | -0.22 | -0.20 |
|  | **0.02** | **0.05** | **0.08** | 0.11 | 0.57 | **0.04** | **0.06** |
| ***Ruminococcus*** | -0.15 | -0.18 | -0.14 | 0.20 | -0.21 | -0.10 | -0.08 |
|  | 0.16 | **0.08** | 0.19 | **0.06** | **0.04** | 0.36 | 0.45 |
| ***Ruminococcus2*** | -0.07 | -0.22 | -0.22 | -0.01 | 0.03 | 0.12 | 0.06 |
|  | 0.52 | **0.04** | **0.03** | 0.89 | 0.79 | 0.24 | 0.58 |
| ***Clostridium* cluster XIV A&B** | -0.15 | -0.03 | -0.01 | -0.01 | 0.07 | -0.05 | 0.02 |
|  | 0.16 | 0.77 | 0.92 | 0.94 | 0.50 | 0.64 | 0.86 |

|  | **BDI** | **STAI Y1** | **STAI Y2** | **PANAS PA** | **PANAS NA** | **PSQI** | **CAR** |
| --- | --- | --- | --- | --- | --- | --- | --- |
| ***Eubacterium*** | -0.21 | -0.25 | -0.24 | 0.09 | -0.04 | 0.04 | -0.06 |
|  | **0.041** | **0.01** | **0.02** | 0.39 | 0.68 | 0.70 | 0.58 |
| ***Coprococcus*** | -0.20 | -0.23 | -0.21 | 0.12 | -0.01 | -0.01 | -0.08 |
|  | **0.06** | **0.03** | **0.04** | 0.26 | 0.95 | 0.96 | 0.42 |
| ***Lactobacillus/Enterococcus*** | -0.18 | -0.24 | -0.22 | -0.02 | -0.14 | -0.03 | -0.13 |
|  | **0.09** | **0.02** | **0.03** | 0.84 | 0.19 | 0.81 | 0.21 |
| ***Lactococcus*** | -0.17 | -0.23 | -0.19 | 0.17 | -0.23 | -0.05 | -0.02 |
|  | 0.10 | **0.03** | **0.07** | 0.11 | **0.03** | 0.64 | 0.86 |
| ***Anaerostipes*** | -0.22 | -0.24 | -0.19 | 0.00 | -0.05 | -0.01 | -0.01 |
|  | **0.04** | **0.02** | **0.06** | 0.99 | 0.61 | 0.95 | 0.95 |
| ***Akkermansia*** | -0.06 | -0.21 | -0.23 | 0.00 | -0.18 | -0.04 | -0.11 |
|  | 0.54 | **0.046** | **0.03** | 0.98 | **0.08** | 0.69 | 0.30 |
| ***Blautia*** | -0.22 | -0.28 | -0.27 | 0.14 | -0.09 | 0.04 | -0.02 |
|  | **0.03** | **0.007** | **0.01** | 0.18 | 0.37 | 0.69 | 0.82 |
| ***Desulfovibrio*** | -0.03 | -0.04 | -0.13 | 0.22 | -0.19 | -0.09 | -0.06 |
|  | 0.80 | 0.67 | 0.22 | **0.04** | **0.07** | 0.37 | 0.55 |
| ***Lachnospiraceae incertae sedis*** | -0.22 | -0.09 | -0.11 | 0.03 | -0.14 | -0.14 | -0.20 |
|  | **0.04** | 0.38 | 0.31 | 0.77 | 0.19 | 0.17 | **0.05** |
| ***Dorea*** | -0.22 | -0.26 | -0.25 | 0.15 | -0.08 | -0.03 | -0.09 |
|  | **0.04** | **0.01** | **0.02** | 0.14 | 0.46 | 0.76 | 0.41 |
| ***Collinsella*** | -0.15 | -0.09 | -0.14 | -0.07 | -0.11 | 0.06 | -0.13 |
|  | 0.15 | 0.37 | 0.19 | 0.49 | 0.29 | 0.58 | 0.21 |
| ***Flavonifractor*** | -0.21 | -0.17 | -0.14 | 0.12 | 0.03 | -0.10 | -0.12 |
|  | **0.04** | 0.11 | 0.19 | 0.27 | 0.77 | 0.34 | 0.27 |
| ***Gemmiger*** | -0.18 | -0.16 | -0.16 | 0.14 | -0.07 | -0.02 | -0.21 |
|  | **0.09** | 0.14 | 0.13 | 0.18 | 0.51 | 0.85 | **0.049** |

Bacterial taxon-mood state interactions from the entire cohort. Pairwise correlations between bacterial taxon and mood states fold change data were calculated using a Spearman’s rank correlation (two sided adjusted for using FDR). Taxa- mood state correlations ranged from -1 to 1 (negative to positive). Red text indicates adjusted *P* (*Q*) values ≤ 0.05; Blue text indicates adjusted *P* (*Q*) values ≥ 0.05 - ≤ 0.07; Green text indicates adjusted *P* (*Q*) values ≥ 0.07 - ≤ 0.09 (grey rows). **Keywords:** BDI = Beck’s Depression Inventory; STAI Y1 and Y2 = State Trait Anxiety Inventory; PANAS = Positive and Negative Affect Schedule – PA = Positive Affect; NA = Negative Affect; CAR = Cortisol Awakening Response; PSQI = Pittsburgh Sleep Quality Index

**Bacterial taxa-taxa correlation matrix**

|  | ***Bifidobacterium*** | ***Bacteroides*** | ***Prevotella*** | ***Alistipes*** | ***Roseburia*** | ***Faecalibacterium prausnitzii*** | ***Rumincoccus*** | ***Ruminococcus2*** | ***Clostridium* Cluster XIV A&B** | ***Eubacterium*** | ***Coprococcus*** | ***Lactobacillus/Enterococcus*** | ***Lactococcus*** | ***Anaerostipes*** | ***Akkermansia*** | ***Blautia*** | ***Desulfovibrio*** | ***Lachnospiraceae incertae sedis*** | ***Dorea*** | ***Collinsella*** | ***Flavonifractor*** | ***Gemmiger*** |
| --- | --- | --- | --- | --- | --- | --- | --- | --- | --- | --- | --- | --- | --- | --- | --- | --- | --- | --- | --- | --- | --- | --- |
| ***Bifidobacterium*** | 1.00 | -0.10 | -0.16 | 0.05 | 0.18 | 0.19 | 0.14 | 0.27 | 0.10 | 0.57 | 0.55 | 0.20 | 0.12 | 0.49 | 0.15 | 0.48 | 0.07 | 0.26 | 0.54 | 0.53 | -0.04 | 0.53 |
|  | 1.00 | 0.35 | 0.14 | 0.66 | 0.09 | **0.07** | 0.18 | **0.01** | 0.33 | 4.03x10^-09^ | **1.76x10^-08^** | **0.05** | 0.26 | **9.43x10^-07^** | 0.16 | **1.59x10^-06^** | 0.51 | **0.01** | **2.34x10^-08^** | **4.70x10^-08^** | 0.72 | **6.00x10^-08^** |
| ***Bacteroides*** | -0.098 | 1.00 | -0.04 | 0.746 | 0.324 | 0.376 | 0.29 | -0.03 | 0.507 | -0.11 | -0.14 | 0.13 | 0.34 | -0.14 | 0.09 | -0.17 | 0.08 | 0.24 | 0.00 | -0.03 | 0.71 | 0.16 |
|  | 0.351 | 1.00 | 0.74 | **1.14x10^-14^** | **0.002** | 2.12x10^-04^ | **0.005** | 0.79 | **2.51x10^-07^** | 0.29 | 0.18 | 0.20 | **0.001** | 0.18 | 0.39 | 0.11 | 0.44 | **0.02** | 0.99 | 0.78 | **3.28x10^-15^** | 0.13 |
| ***Prevotella*** | -0.16 | -0.04 | 1.00 | -0.10 | -0.12 | -0.210 | -0.176 | 0.079 | -0.096 | -0.143 | -0.103 | 0.045 | -0.154 | -0.131 | 0.110 | -0.104 | -0.093 | -0.120 | -0.094 | -0.122 | -0.053 | -0.236 |
|  | 0.14 | 0.74 | 1.00 | 0.33 | 0.24 | **0.045** | 0.094 | 0.454 | 0.365 | 0.175 | 0.331 | 0.673 | 0.142 | 0.215 | 0.298 | 0.324 | 0.378 | 0.253 | 0.374 | 0.248 | 0.615 | **0.024** |
| ***Alistipes*** | 0.05 | 0.75 | -0.10 | 1.00 | 0.26 | 0.302 | 0.428 | 0.034 | 0.461 | 0.079 | 0.057 | 0.016 | 0.482 | 0.080 | 0.204 | 0.022 | 0.270 | 0.247 | 0.066 | 0.005 | 0.649 | 0.307 |
|  | 0.66 | **1.14x10^-14^** | 0.33 | 1.00 | 0.01 | **0.003** | **2.08x10^-05^** | 0.75 | **3.82x10^-06^** | 0.46 | 0.59 | 0.88 | **1.15x10^-06^** | 0.45 | **0.05** | 0.83 | **0.01** | **0.02** | 0.53 | 0.97 | **2.76x10^-12^** | **0.003** |

|  | ***Bifidobacterium*** | ***Bacteroides*** | ***Prevotella*** | ***Alistipes*** | ***Roseburia*** | ***Faecalibacterium prausnitzii*** | ***Rumincoccus*** | ***Ruminococcus2*** | ***Clostridium* Cluster XIV A&B** | ***Eubacterium*** | ***Coprococcus*** | ***Lactobacillus/Enterococcus*** | ***Lactococcus*** | ***Anaerostipes*** | ***Akkermansia*** | ***Blautia*** | ***Desulfovibrio*** | ***Lachnospiraceae incertae sedis*** | ***Dorea*** | ***Collinsella*** | ***Flavonifractor*** | ***Gemmiger*** |
| --- | --- | --- | --- | --- | --- | --- | --- | --- | --- | --- | --- | --- | --- | --- | --- | --- | --- | --- | --- | --- | --- | --- |
| ***Roseburia*** | 0.18 | 0.32 | -0.12 | 0.26 | 1.00 | 0.42 | 0.47 | 0.17 | 0.40 | 0.23 | 0.18 | -0.08 | 0.39 | 0.12 | 0.04 | 0.28 | 0.16 | 0.26 | 0.30 | 0.01 | 0.14 | 0.46 |
|  | 0.09 | **0.002** | 0.24 | **0.01** | 1.00 | **2.42x10^-05^** | **2.65x10^-05^** | 0.11 | **7.24x10^-05^** | **0.03** | 0.09 | 0.42 | **0.0001** | 0.24 | 0.68 | **0.01** | 0.14 | **0.01** | **0.004** | 0.89 | 0.19 | **4.76x10^-06^** |
| ***Faecalibacterium prausnitzii*** | 0.19 | 0.38 | -0.21 | 0.30 | 0.42 | 1.00 | 0.38 | 0.18 | 0.48 | 0.18 | 0.14 | 0.00 | 0.32 | 0.09 | -0.12 | 0.22 | 0.00 | 0.34 | 0.15 | -0.06 | 0.28 | 0.38 |
|  | **0.07** | **2.12x10^-04^** | **0.04** | **0.003** | **2.42x10^-05^** | 1.00 | **0.0002** | 0.09 | **1.10x10^-06^** | 0.09 | 0.18 | 0.97 | **0.002** | 0.39 | 0.24 | **0.04** | 0.96 | **0.001** | 0.15 | 0.59 | **0.01** | **0.0002** |
| ***Ruminococcus*** | 0.14 | 0.29 | -0.18 | 0.43 | 0.47 | 0.38 | 1.00 | 0.18 | 0.34 | 0.23 | 0.19 | -0.06 | 0.91 | 0.04 | 0.03 | 0.36 | 0.23 | 0.38 | 0.23 | -0.03 | 0.25 | 0.44 |
|  | 0.18 | **0.01** | 0.09 | **2.08x10^-05^** | **2.65x10^-05^** | **0.0002** | 1.00 | 0.09 | **0.001** | **0.03** | **0.07** | 0.54 | **1.14x10^-35^** | 0.72 | 0.77 | **0.0004** | **0.03** | **0.0001** | **0.03** | 0.81 | **0.02** | **1.31x10^-05^** |
| ***Ruminococcus2*** | 0.27 | -0.03 | 0.08 | 0.03 | 0.17 | 0.177 | 0.18 | 1.0 | 0.176 | 0.61 | 0.59 | 0.01 | 0.23 | 0.38 | 0.19 | 0.68 | 0.15 | 0.20 | 0.49 | 0.28 | 0.11 | 0.39 |
|  | **0.01** | 0.79 | 0.45 | 0.75 | 0.11 | 0.092 | 0.09 | 1.0 | 0.09 | **1.38x10^-10^** | **6.15x10^-10^** | 0.93 | **0.03** | **0.0002** | **0.08** | **8.33x10^-14^** | 0.16 | **0.05** | **6.98x10^-07^** | **0.01** | 0.30 | **0.0001** |

|  | ***Bifidobacterium*** | ***Bacteroides*** | ***Prevotella*** | ***Alistipes*** | ***Roseburia*** | ***Faecalibacterium prausnitzii*** | ***Rumincoccus*** | ***Ruminococcus2*** | ***Clostridium* Cluster XIV A&B** | ***Eubacterium*** | ***Coprococcus*** | ***Lactobacillus/Enterococcus*** | ***Lactococcus*** | ***Anaerostipes*** | ***Akkermansia*** | ***Blautia*** | ***Desulfovibrio*** | ***Lachnospiraceae incertae sedis*** | ***Dorea*** | ***Collinsella*** | ***Flavonifractor*** | ***Gemmiger*** |
| --- | --- | --- | --- | --- | --- | --- | --- | --- | --- | --- | --- | --- | --- | --- | --- | --- | --- | --- | --- | --- | --- | --- |
| ***Clostridium* cluster XIV A&B** | 0.10 | 0.51 | -0.10 | 0.46 | 0.40 | 0.483 | 0.34 | 0.2 | 1.00 | 0.13 | 0.12 | 0.02 | 0.29 | 0.06 | -0.05 | 0.15 | 0.12 | 0.45 | 0.22 | 0.11 | 0.40 | 0.27 |
|  | 0.33 | **2.51x10^-07^** | 0.36 | **3.82x10^-06^** | **7.24x10^-05^** | **1.10x10^-06^** | **0.001** | 0.1 | 1.00 | 0.22 | 0.26 | 0.85 | **0.004** | 0.60 | 0.63 | 0.15 | 0.24 | **7.64x10^-06^** | **0.03** | 0.31 | **8.55x10^-05^** | **0.01** |
| ***Eubacterium*** | 0.57 | -0.11 | -0.14 | 0.08 | 0.23 | 0.18 | 0.23 | 0.6 | 0.13 | 1.00 | 0.98 | 0.09 | 0.22 | 0.66 | 0.21 | 0.77 | 0.16 | 0.25 | 0.60 | 0.42 | 0.04 | 0.61 |
|  | **4.03x10^-09^** | 0.29 | 0.17 | 0.46 | **0.03** | 0.09 | **0.03** | **1.38x10^-10^** | 0.22 | 1.00 | **1.30x10^-64^** | 0.37 | **0.03** | **6.19x10^-13^** | **0.049** | **1.54x10^-19^** | 0.12 | **0.02** | **2.03x10^-10^** | **3.18x10^-05^** | 0.68 | **1.29x10^-10^** |
| ***Coprococcus*** | 0.55 | -0.14 | -0.10 | 0.06 | 0.18 | 0.14 | 0.19 | 0.59 | 0.12 | 0.98 | 1.00 | 0.10 | 0.18 | 0.67 | 0.18 | 0.75 | 0.19 | 0.23 | 0.60 | 0.40 | 0.04 | 0.55 |
|  | **1.76x10^-08^** | 0.18 | 0.33 | 0.59 | 0.09 | 0.18 | **0.07** | **6.15x10^-10^** | 0.26 | **1.30x10^-64^** | 1.00 | 0.35 | **0.08** | **2.42x10^-13^** | **0.08** | **7.96x10^-18^** | **0.08** | **0.03** | **2.85x10^-10^** | **7.68x10^-05^** | 0.70 | **1.85x10^-08^** |
| ***Lactobacillus/Enterococcus*** | 0.20 | 0.13 | 0.04 | 0.02 | -0.08 | 0.00 | -0.06 | 0.01 | 0.02 | 0.09 | 0.10 | 1.00 | -0.01 | 0.09 | 0.03 | 0.07 | -0.12 | -0.03 | 0.14 | 0.07 | 0.10 | 0.06 |
|  | **0.05** | 0.20 | 0.67 | 0.88 | 0.42 | 0.97 | 0.54 | 0.93 | 0.85 | 0.37 | 0.35 | 1.00 | 0.93 | 0.37 | 0.80 | 0.52 | 0.25 | 0.74 | 0.17 | 0.52 | 0.34 | 0.56 |

|  | ***Bifidobacterium*** | ***Bacteroides*** | ***Prevotella*** | ***Alistipes*** | ***Roseburia*** | ***Faecalibacterium prausnitzii*** | ***Rumincoccus*** | ***Ruminococcus2*** | ***Clostridium* Cluster XIV A&B** | ***Eubacterium*** | ***Coprococcus*** | ***Lactobacillus/Enterococcus*** | ***Lactococcus*** | ***Anaerostipes*** | ***Akkermansia*** | ***Blautia*** | ***Desulfovibrio*** | ***Lachnospiraceae incertae sedis*** | ***Dorea*** | ***Collinsella*** | ***Flavonifractor*** | ***Gemmiger*** |
| --- | --- | --- | --- | --- | --- | --- | --- | --- | --- | --- | --- | --- | --- | --- | --- | --- | --- | --- | --- | --- | --- | --- |
| ***Lactococcus*** | 0.12 | 0.34 | -0.15 | 0.48 | 0.39 | 0.32 | 0.91 | 0.23 | 0.29 | 0.22 | 0.18 | -0.01 | 1.00 | 0.06 | 0.04 | 0.32 | 0.23 | 0.36 | 0.24 | -0.06 | 0.30 | 0.43 |
|  | 0.26 | **0.001** | 0.14 | **1.15x10^-06^** | **0.0001** | **0.002** | **1.14x10^-35^** | **0.03** | **0.004** | **0.03** | **0.08** | 0.93 | 1.00 | 0.56 | 0.71 | **0.001** | **0.03** | **0.00** | **0.02** | 0.54 | **0.004** | **2.35x10^-05^** |
| ***Anaerostipes*** | 0.49 | -0.14 | -0.13 | 0.08 | 0.12 | 0.09 | 0.04 | 0.38 | 0.06 | 0.66 | 0.67 | 0.09 | 0.06 | 1.00 | 0.15 | 0.52 | 0.07 | 0.16 | 0.40 | 0.27 | -0.01 | 0.36 |
|  | **9.43x10^-07^** | 0.18 | 0.21 | 0.45 | 0.24 | 0.39 | 0.72 | **0.0002** | 0.60 | **6.19x10^-13^** | **2.42x10^-13^** | 0.37 | 0.56 | 1.00 | 0.14 | **1.45x10^-07^** | 0.48 | 0.13 | **6.67x10^-05^** | **0.01** | 0.89 | 0.54 |
| ***Akkermansia*** | 0.15 | 0.09 | 0.11 | 0.20 | 0.04 | -0.12 | 0.03 | 0.19 | -0.05 | 0.21 | 0.18 | 0.03 | 0.04 | 0.15 | 1.00 | 0.14 | 0.23 | 0.05 | 0.05 | 0.12 | 0.14 | 0.06 |
|  | 0.16 | 0.39 | 0.30 | **0.05** | 0.68 | 0.24 | 0.77 | **0.08** | 0.63 | **0.049** | 0.08 | 0.80 | 0.71 | 0.14 | 1.00 | 0.19 | **0.02** | 0.61 | 0.61 | 0.25 | 0.18 | 0.54 |
| ***Blautia*** | 0.48 | -0.17 | -0.10 | 0.02 | 0.28 | 0.22 | 0.36 | 0.68 | 0.15 | 0.77 | 0.75 | 0.07 | 0.32 | 0.52 | 0.14 | 1.00 | 0.24 | 0.19 | 0.70 | 0.40 | 0.02 | 0.57 |
|  | **1.59x10^-06^** | 0.11 | 0.32 | 0.83 | **0.01** | **0.04** | **0.0004** | **8.33x10^-14^** | 0.15 | **1.54x10^-19^** | **7.96x10^-18^** | 0.52 | **0.002** | **1.45x10^-07^** | 0.19 | 1.00 | **0.02** | **0.07** | **9.82x10^-15^** | **7.32x10^-05^** | 0.88 | **2.39x10^-09^** |

|  | ***Bifidobacterium*** | ***Bacteroides*** | ***Prevotella*** | ***Alistipes*** | ***Roseburia*** | ***Faecalibacterium prausnitzii*** | ***Rumincoccus*** | ***Ruminococcus2*** | ***Clostridium* Cluster XIV A&B** | ***Eubacterium*** | ***Coprococcus*** | ***Lactobacillus/Enterococcus*** | ***Lactococcus*** | ***Anaerostipes*** | ***Akkermansia*** | ***Blautia*** | ***Desulfovibrio*** | ***Lachnospiraceae incertae sedis*** | ***Dorea*** | ***Collinsella*** | ***Flavonifractor*** | ***Gemmiger*** |
| --- | --- | --- | --- | --- | --- | --- | --- | --- | --- | --- | --- | --- | --- | --- | --- | --- | --- | --- | --- | --- | --- | --- |
| ***Desulfovibrio*** | 0.07 | 0.08 | -0.09 | 0.27 | 0.16 | 0.00 | 0.23 | 0.15 | 0.12 | 0.16 | 0.19 | -0.12 | 0.23 | 0.07 | 0.23 | 0.24 | 1.00 | 0.12 | 0.09 | 0.01 | 0.08 | 0.19 |
|  | 0.51 | 0.44 | 0.38 | **0.01** | 0.14 | 0.96 | **0.03** | 0.16 | 0.24 | 0.12 | **0.08** | 0.25 | **0.03** | 0.48 | **0.02** | **0.02** | 1.00 | 0.26 | 0.39 | 0.95 | 0.48 | **0.07** |
| ***Lachnospiraceae incertae sedis*** | 0.26 | 0.24 | -0.12 | 0.25 | 0.26 | 0.34 | 0.38 | 0.20 | 0.45 | 0.25 | 0.23 | -0.03 | 0.36 | 0.16 | 0.05 | 0.19 | 0.12 | 1.00 | 0.22 | 0.10 | 0.16 | 0.24 |
|  | **0.01** | **0.02** | 0.25 | **0.02** | **0.01** | **0.001** | **0.0002** | **0.05** | **7.64x10^-06^** | **0.02** | **0.03** | 0.74 | **0.0004** | 0.13 | 0.61 | **0.07** | 0.26 | 1.00 | **0.04** | 0.36 | 0.14 | **0.02** |
| ***Dorea*** | 0.54 | 0.00 | -0.09 | 0.07 | 0.30 | 0.15 | 0.23 | 0.49 | 0.22 | 0.60 | 0.60 | 0.14 | **0.24** | 0.40 | 0.05 | 0.70 | 0.09 | 0.22 | 1.00 | 0.45 | 0.10 | 0.56 |
|  | **2.34x10^-08^** | 0.99 | 0.37 | 0.53 | **0.004** | 0.15 | **0.03** | **6.98x10^-07^** | **0.03** | **2.03x10^-10^** | **2.85x10^-10^** | 0.17 | **0.02** | **6.67x10^-05^** | 0.61 | **9.82x10^-15^** | 0.39 | **0.04** | 1.00 | **6.49x10^-06^** | 0.34 | **6.26x10^-09^** |
| ***Collinsella*** | 0.53 | -0.03 | -0.12 | 0.00 | 0.01 | -0.06 | -0.03 | 0.28 | 0.11 | 0.42 | 0.40 | 0.07 | -0.06 | 0.27 | 0.12 | 0.40 | 0.01 | 0.10 | 0.45 | 1.00 | 0.09 | 0.33 |
|  | **4.70x10^-08^** | 0.78 | 0.25 | 0.97 | 0.89 | 0.59 | 0.81 | **0.01** | 0.31 | **3.18x10^-05^** | **7.68x10^-05^** | 0.52 | 0.54 | **0.01** | 0.25 | **7.32x10^-05^** | 0.95 | 0.36 | **6.49x10^-06^** | 1.00 | 0.38 | **0.001** |

|  | ***Bifidobacterium*** | ***Bacteroides*** | ***Prevotella*** | ***Alistipes*** | ***Roseburia*** | ***Faecalibacterium prausnitzii*** | ***Rumincoccus*** | ***Ruminococcus2*** | ***Clostridium* Cluster XIV A&B** | ***Eubacterium*** | ***Coprococcus*** | ***Lactobacillus/Enterococcus*** | ***Lactococcus*** | ***Anaerostipes*** | ***Akkermansia*** | ***Blautia*** | ***Desulfovibrio*** | ***Lachnospiraceae incertae sedis*** | ***Dorea*** | ***Collinsella*** | ***Flavonifractor*** | ***Gemmiger*** |
| --- | --- | --- | --- | --- | --- | --- | --- | --- | --- | --- | --- | --- | --- | --- | --- | --- | --- | --- | --- | --- | --- | --- |
| ***Flavonifractor*** | -0.04 | 0.71 | -0.05 | 0.65 | 0.14 | 0.28 | 0.25 | 0.11 | 0.40 | 0.04 | 0.04 | 0.10 | 0.30 | -0.01 | 0.14 | 0.02 | 0.08 | 0.16 | 0.10 | 0.09 | 1.00 | 0.19 |
|  | 0.72 | **3.28x10^-15^** | 0.62 | **2.76x10^-12^** | 0.19 | **0.01** | **0.02** | 0.30 | **8.55x10^-05^** | 0.68 | 0.70 | 0.34 | **0.004** | 0.89 | 0.18 | 0.88 | 0.48 | 0.14 | 0.34 | 0.38 | 1.00 | **0.07** |
| ***Gemmiger*** | 0.53 | 0.16 | -0.24 | 0.31 | 0.46 | 0.38 | 0.44 | 0.39 | 0.27 | 0.61 | 0.55 | 0.06 | 0.43 | 0.36 | 0.06 | 0.57 | 0.19 | 0.24 | 0.56 | 0.33 | 0.19 | 1.00 |
|  | **6.00x10^-08^** | 0.13 | **0.02** | **0.003** | **4.76x10^-06^** | **0.0002** | **1.31x10^-05^** | **0.0001** | **0.01** | **1.29x10^-10^** | **1.85x10^-08^** | 0.56 | **2.35x10^-05^** | **0.0004** | 0.54 | **2.39x10^-09^** | 0.07 | **0.02** | **6.26x10^-09^** | **0.001** | **0.07** | 1.00 |

Bacterial taxa-taxa interactions from the entire cohort. Pairwise correlations between bacterial taxon using fold change data were calculated using a Spearman’s rank correlation (two sided adjusted for using FDR). Taxa- mood state correlations ranged from -1 to 1 (negative to positive). Red text indicates adjusted *P* (*Q*) values ≤ 0.05; Blue text indicates adjusted *P* (*Q*) values ≥ 0.05 - ≤ 0.07; Green text indicates adjusted *P* (*Q*) values ≥ 0.07 - ≤ 0.09 (grey rows).
